# Metabolic remodeling of microorganisms by mobile genetic elements alters mutualistic community composition

**DOI:** 10.1128/msystems.00144-25

**Published:** 2025-08-15

**Authors:** Ave T. Bisesi, Ross P. Carlson, Lachlan Cotner, William R. Harcombe

**Affiliations:** 1 Department of Ecology, Evolution and Behavior, University of Minnesota 172734 , St. Paul, Minnesota, USA; 2 Department of Chemical and Biological Engineering, Montana State University 33052, Bozeman, Montana, USA; 3 Center for Biofilm Engineering, Montana State University 827241, Bozeman, Montana, USA; 4 BioTechnology Institute, University of Minnesota, St. Paul, Minnesota, USA; University of Michigan, Ann Arbor, Michigan, USA

**Keywords:** filamentous bacterial virus, conjugation, microbial communities, metabolic modeling, plasmid

## Abstract

Mobile genetic elements (MGEs) are ubiquitous in prokaryotes and exert significant influence on microbial communities, in part through their effects on host metabolism. While some MGEs directly alter host metabolism by introducing novel enzymes, all MGEs can indirectly change bacterial metabolism by redirecting intracellular host resources away from native bacterial processes toward MGE production. As a result, even when MGEs do not provide new metabolic functions, their carriage can influence host traits such as growth rate, nutrient uptake, and waste excretion, with consequences for how bacteria contribute to community and ecosystem functions. However, there are few empirical tests of how the indirect metabolic consequences of MGE carriage shape interactions between host and non-host bacterial species. We integrated genome-scale metabolic modeling with an *in vitro* obligate cross-feeding system to investigate the metabolic consequences of two MGEs in *Escherichia coli*: the conjugative plasmid F128 and the filamentous phage M13. We examined the impact of these MGEs on interactions between bacteria in a multispecies cross-feeding community composed of *E. coli*, *Salmonella enterica,* and *Methylobacterium extorquens*. Both modeling and *in vitro* experiments suggested that MGE carriage can change the growth rate and excretion profile of *E. coli*. We also found that indirect changes to host metabolism induced by our MGEs increased the density of cross-feeding species. Our work emphasizes that microbes carrying MGEs can have different metabolisms than MGE-free cells, even when MGEs do not encode metabolic enzymes, and demonstrates that these metabolic shifts can have significant consequences for microbial community structure and function.

**IMPORTANCE:**

Mobile genetic elements (MGEs) often shape the structure and function of microbial communities by influencing the metabolism of bacterial cells. Though some MGEs change metabolism directly by transferring genetic material that provides access to novel niche space, all MGEs should alter host metabolism indirectly to some degree by shifting intracellular metabolic processes toward MGE replication. This study uses a combination of flux balance analysis and an *in vitro* system consisting of *Escherichia coli*, *Salmonella enterica*, *Methylobacterium extorquens*, and two MGEs in *E. coli* to investigate how MGEs change the community contributions of their hosts via metabolic conflict alone. Flux balance analysis suggests that MGEs can change intracellular demand for different metabolic processes, leading to shifts in the identities and concentrations of compounds that hosts externalize into the environment. This finding is supported by experimental results and extends our understanding of how MGEs shape the structure and function of microbial communities.

## INTRODUCTION

Mobile genetic elements (MGEs), such as bacteriophage (phage) and plasmids, serve as important drivers of microbial community composition and function in large part by altering host metabolism (1, 2). Many MGEs impact host metabolism directly by introducing new functions or physiological niches to host cells through horizontal gene transfer, which can enable new interactions between microbial species, such as competitor-inhibiting toxin production or mutualistic cross-feeding (3, 4). Access to these phenotypes can have significant consequences for the coexistence and relative abundances of bacterial species (3–6), in addition to impacting the plants, animals, or humans associated with a microbial community by mediating traits like microbial virulence or antibiotic resistance (1, 7–9). However, while only a subset of MGEs carry metabolic genes that enable novel functions in their hosts, all MGEs have the potential to disrupt microbial metabolism indirectly through the costs of carriage. MGEs generally rely on host machinery for replication, transcription, and translation, redirecting intracellular resources away from host processes toward MGE production (1, 8, 10, 11). Though the carriage (i.e., presence in a cell) of an MGE may not necessarily result in a high metabolic burden (i.e., a large proportion of intracellular resources directed away from core host processes), this type of metabolic conflict has the potential to rewire host metabolic pathways, modulating phenotypes like bacterial growth rate and nutrient requirements (10, 12–16). Because interactions between microbes are built on a metabolic foundation (12, 17–22), MGEs could have substantial community-wide consequences if the cost of carriage alters key metabolic traits like host nutrient usage, waste excretion, or growth rate. However, it is not clear how different types of MGEs impact microbial community composition and function through metabolic remodeling alone. Given the ubiquity of MGEs in natural systems (23, 24), it is essential to better understand how the metabolic costs of MGE carriage structure microbial communities across environments.

Plasmids are a class of well-studied MGE that can drive both ecological and evolutionary dynamics in bacterial communities (25). These extrachromosomal DNA elements are transmitted between host cells both vertically through cell division and horizontally through mechanisms like conjugation or transformation (7, 26). By facilitating the spread of beneficial genes (1), plasmids often form sustained associations with bacterial strains (7), structure microbial communities in diverse environments by mediating traits like biofilm formation (27, 28), and contribute significantly to clinically relevant crises such as the emergence of antibiotic resistance in human-associated bacterial pathogens (25). However, though they confer benefits in some environments, plasmids also parasitize the intracellular resources and machinery of host cells, meaning that the costs of their carriage can be substantial and result in poor growth of plasmid-positive strains (25). These costs can exist even under environmental conditions selectively favorable for traits carried by the plasmid (29) and are often driven by conflicts of gene expression that emerge primarily at the level of translation (25). Generally, this metabolic burden is sustained until the plasmid is lost during cell division (30) or some kind of compensatory evolution occurs (31). In some cases, the metabolic costs of plasmid acquisition are substantial enough to protect hosts against antibiotics without conferring resistance genes by inducing host stress responses (32). Highly diverse plasmids can even drive convergent indirect metabolic responses, as has been shown in *Pseudomonas aeruginosa* (33). Because their carriage has the potential to shape host growth rate and nutrient exchange, even when they do not directly contribute to changes in host metabolism via horizontal gene transfer, investigating the metabolic costs of plasmid acquisition and transmission may further our understanding of how MGEs shape microbial communities through indirect changes to host metabolism.

Phages are another type of ecologically consequential MGE with both direct and indirect consequences for host metabolism. The ability of phage to reduce host densities, free nutrients via lysis, and promote bacterial diversity through mechanisms such as “Kill-the-Winner” (10, 34, 35) makes them central to ecosystem processes like the viral shunt and shuttle in both aquatic (36–38) and terrestrial (39) environments. However, unlike lytic and lysogenic phage, filamentous phage can shape microbial communities through the indirect process of chronic metabolic remodeling alone. This is because filamentous phage are carried by hosts either episomally or as prophage (40) and closely mirror plasmids in their capacity for horizontal and vertical transmission. Importantly, filamentous phage do not induce lysis like other forms of phage and instead extrude continually from living cells, allowing for associations between hosts and phage that are sustained both within and across microbial generations (41, 42). Moreover, the continual activity of filamentous phage means that the metabolic burden they impose is more akin to that of plasmid expression than other MGEs such as dormant lysogenic phage, which may only express a handful of genes during lysogeny (11). Symbiotic relationships can also arise between filamentous phage and their hosts (43), either through direct introduction of novel functions or through indirect metabolic remodeling by phage infection that alters host growth rate and other traits in adaptive ways. The metabolic consequences of filamentous phage are reflected in their importance across microbial systems. For example, they have been implicated in the delayed healing of infected wounds (44), improved biofilm formation (45) and colonization of epithelial cells (46), and reduced diffusion of antibiotics (47). Understanding how MGE carriage shapes the composition and function of microbial communities through metabolic conflict between MGE production and bacterial replication, therefore, requires close attention to the metabolic remodeling induced by these plasmid-like phages.

**Fig 1:**
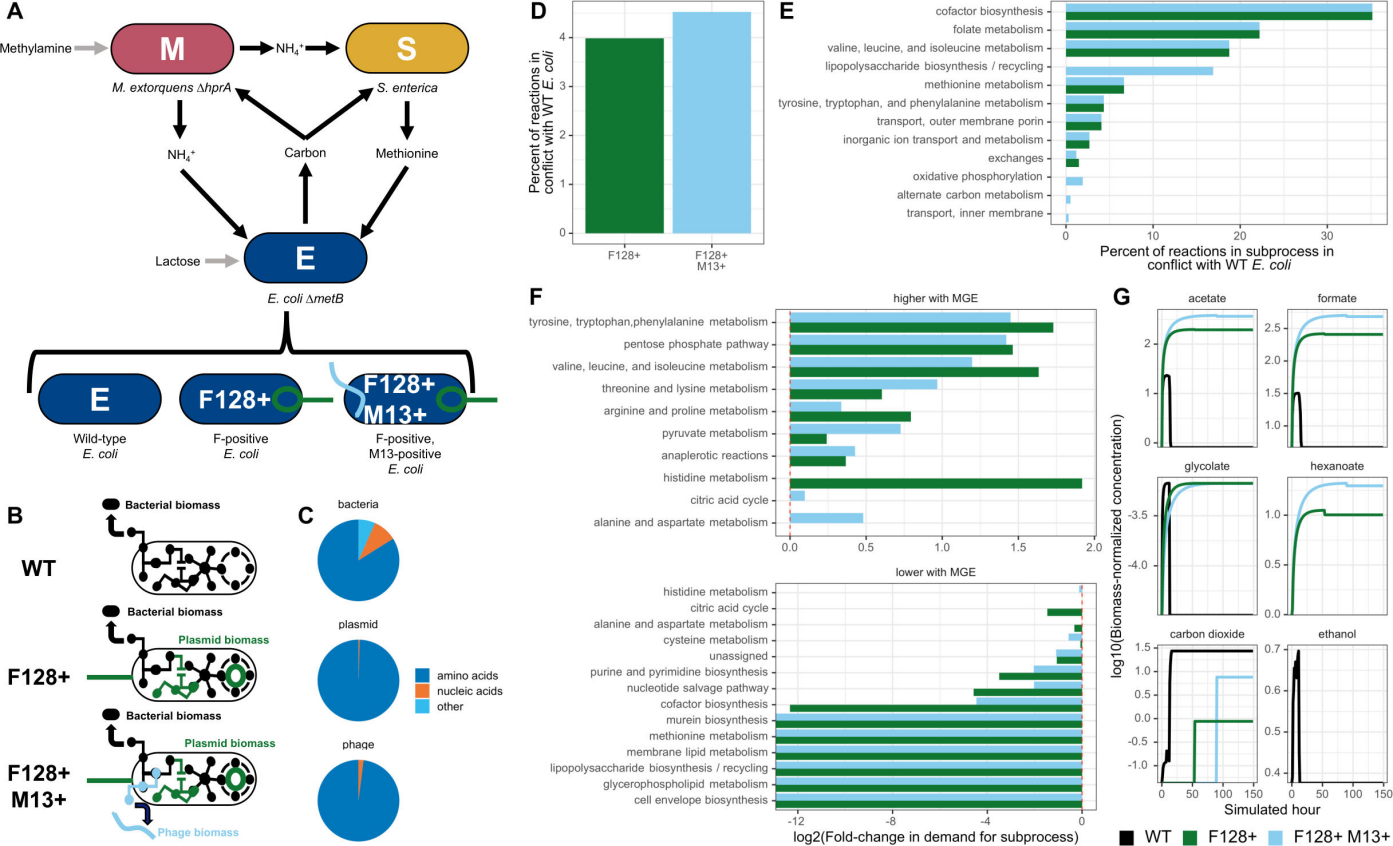
Genome-scale metabolic modeling predicts that mobile genetic element carriage increases demand for amino acids, decreases demand for host-specific processes, and limits reuptake of waste products. (**A**) Schematic of a synthetic cross-feeding microbial system with associated carriage statuses of *E. coli ∆metB*. (**B**) Schematic of *E. coli ∆metB* metabolic models used for FBA analyses. Models were forced to produce either only bacterial biomass or bacterial biomass in addition to plasmid and/or phage biomass. Both plasmid and phage biomass served as nutrient sinks in metabolic models; extracellular transport of phage particles was not modeled. (**C**) Pie charts displaying the ratios of the different components of biomass reactions for hosts, phage, and plasmids used in FBA simulations. (**D**) Percent of total metabolic reactions in the *E. coli ∆metB* metabolic system with non-overlapping flux ranges (FVA) when MGE biomass is optimized versus when host biomass is optimized. F128+ M13+ simulations were run with a lower bound of 0.9 mmol gDW^−1^ hr^−1^ on plasmid production while maximizing phage-M13 production. (E) Percent of reactions in each cellular subprocess with non-overlapping flux ranges (FVA) when MGE biomass is optimized versus when host biomass is optimized. F128+ M13+ simulations were run with a lower bound of 0.9 mmol gDW^−1^ hr^−1^ on plasmid production while maximizing phage-M13 production. (**F**) Log2(fold-change) in flux magnitude (i.e., demand) for all reactions in each cellular subprocess (pFBA) when MGE biomass is optimized versus when host biomass is optimized. Positive values indicate that there is more demand for a subprocess when MGE biomass is optimized. Negative values indicate that there is less demand for a subprocess when MGE biomass is optimized. F128+ M13+ simulations were run with a lower bound of 0.9 mmol gDW^−1^ hr^−1^ on plasmid production while maximizing phage-M13 production. Only cellular subprocesses where the absolute value of the log2(fold-change) was greater than 0.5 for at least one carriage status are shown. (**G**) Simulated log10(mmol gDW^−1^) of key externalized metabolites during monoculture growth of *E. coli ∆metB* with various carriage statuses (dFBA). Lactose is the only carbon source provided in the initial media. F128+ was modeled with a lower bound on plasmid production of 0.9 mmol gDW^−1^ hr^−1^, and F128+ M13+ was modeled with a lower bound of 0.9 mmol gDW^−1^ hr^−1^ on plasmid production and 0.06 mmol gDW^−1^ hr^−1^ on phage production.

To consider the impact of metabolic conflict between hosts and MGEs on microbial communities, we used a combination of a synthetic system of three cross-feeding bacterial species—*Escherichia coli*, *Salmonella enterica*, and *Methylobacterium extorquens* (Fig. 1A)—and a genome-scale metabolic modeling approach focused on the nucleotide and amino acid cost of carriage of the conjugative plasmids F128 (plasmid-F128) and the filamentous phage M13 (phage-M13) (Fig. 1B and C). We chose these two MGEs specifically because neither carries metabolic enzymes expected to directly induce selectively relevant new functions in their *E. coli* host, nor should they cause cellular lysis. Instead, we expected that any observed changes to microbial community composition and function should occur primarily due to the metabolic conflict between host cells and MGE production. Furthermore, these two MGEs form a consequential association in nature, given that phage-M13, like related Ff filamentous phages and many other phages, relies on the conjugative pilus produced by plasmid-F128 to gain cellular entry to hosts (40, 41, 48, 49). Carriage of plasmid-F128 (or any plasmid that includes the conjugative pilus machinery) is therefore a prerequisite for phage-M13 infection in most natural systems (40, 41) and should shape the metabolic dynamics of phage-M13 carriage in microbial communities. To test the prediction that metabolic conflict between a host and an MGE can be sufficient to reshape bacterial communities, we compared modeling-predicted changes in host metabolic processes with *in vitro* changes in community composition and function and found that MGE carriage altered the growth rate and metabolic contributions of our *E. coli* host to the microbial community in which it was embedded. Our results underscore the importance of characterizing the metabolic heterogeneity of microbes carrying various MGEs to successfully predict and manage complex microbial communities.

## RESULTS

### Metabolic conflict between *E. coli* and two MGEs is predicted to be driven primarily by demand for amino acids

To predict the consequences of MGE carriage for host metabolic processes, we performed genome-scale metabolic modeling using flux balance analysis (FBA) (50) to consider metabolic conflict between an *E. coli ∆metB* strain and two of its MGEs, the F128 plasmid and the filamentous phage M13 (Fig. 1A). MGE-free *E. coli ∆metB* served as our metabolic “wild-type” (WT), with MGE carriage impacting metabolism relative to that background. Our MGEs were modeled using amino acid and nucleotide costs calculated from their genomic content, forcing the metabolic system of *E. coli ∆metB* to produce MGE biomass in addition to or instead of host biomass (Fig. 1B and C). We followed a modeling approach that has been previously applied to plasmids (51), DNA and RNA-based lytic bacteriophage (52, 53), and human viruses (54). In addition to host biomass, cells were forced to produce either plasmid-F128 biomass (F128+) or both plasmid-F128 and phage-M13 biomass (F128+ M13+). MGE biomass served as a metabolic sink for the intracellular metabolites required for production; though *in vitro* filamentous phage exit the cell continuously without lysis, we did not include extracellular transport of phage in our pseudoreactions.

With our integrated host-MGE metabolic model, we first sought to determine whether differences in host metabolism arose because of MGE carriage. We did so in alignment with existing work (54) by examining our model in (i) a host-optimized state, where the metabolism of *E. coli ∆metB *was optimized to produce only new bacterial biomass and no MGE biomass was produced, (ii) an MGE-optimized state, where the metabolism of *E. coli ∆metB* was optimized to produce new MGE biomass and no bacterial biomass was produced, and (iii) an intermediary state, where the metabolism of *E. coli ∆metB* was optimized for the production of new host biomass, but a minimum lower bound was placed on MGE production. The first two states represented potential metabolic extremes, while we expected the intermediate state to be more representative of the average host cell state during MGE carriage, where bacterial reproduction is possible but significantly constrained by the metabolic demands of producing MGEs (54). When we modeled hosts carrying only the plasmid in an MGE-optimized state, a constant lower bound of 0 was placed on phage-M13 production (lower flux bound on phage pseudoreaction = 0 mmol per gram of dry weight per hour [mmol gDW^−1^ hr^−1^]), while plasmid production was optimized. When we modeled hosts carrying both MGEs in an MGE-optimized state, a constant lower bound was placed on plasmid production (lower flux bound on plasmid pseudoreaction = 0.9 mmol gDW^−1^ hr^−1^), while phage-M13 production was optimized. For each scenario, we investigated the full range of potential FBA solutions by performing flux variability analysis (FVA), parsimonious flux balance analysis (pFBA), and dynamic flux balance analysis (dFBA) using COBRAPy (55) and the Python toolkit for the platform **C**omputation **o**f **M**icrobial **E**cosystems in **T**ime and **S**pace (COMETS) (56).

After obtaining FVA solutions, we identified metabolic sites of conflict by comparing the flux ranges of all reactions predicted by FVA between a host-optimized and MGE-optimized state. Reactions where the flux ranges did not overlap were considered high conflict, given that, by definition, they had no shared solutions between optimization states. Of the 2,585 metabolic reactions in the *E. coli ∆metB* metabolic system, we found that 103 (4.0%) were sites of metabolic conflict when *E. coli ∆metB* carried plasmid-F128 and 117 (4.5%) were sites of metabolic conflict when *E. coli ∆metB* carried both MGEs (Fig. 1D). All the predicted sites of conflict in F128+ except one were shared by F128+ M13+ *E. coli ∆metB*, while 15 reactions were unique to F128+ M13+ *E. coli ∆metB*, consistent with the significant amino acid and nucleotide cost of simultaneously producing plasmid and phage biomass.

We next matched each high-conflict reaction to its corresponding cellular subprocess. We found that three amino acid processes—methionine metabolism; tyrosine, tryptophan, and phenylalanine metabolism; and valine, leucine, and isoleucine metabolism—were disrupted to a similar degree in each carriage status (Fig. 1E). We also saw that carrying both MGEs increased conflict in lipopolysaccharide, oxidative phosphorylation, and inner membrane transport reactions, consistent with the expectation that producing MGE biomass should downregulate many host-specific processes (Fig. 1E). To understand whether these reactions were high conflict due to an increased or decreased demand for those cellular subprocesses during MGE carriage, we quantified the log2(fold-change) in parsimonious flux for all reactions in each subprocess in an MGE-optimized relative to a host-optimized state (Fig. 1F). Carriage increased demand for several amino acid biosynthesis processes regardless of carriage state, as well as the pentose phosphate pathway, pyruvate metabolism, and anaplerotic reactions (Fig. 1F). By contrast, carriage reduced demand for many host-specific processes, particularly cofactor biosynthesis, murein biosynthesis, and other membrane biosynthesis pathways (Fig. 1F), given that producing MGE particles did not require compounds like lipids. Taken together, these findings underscore that nucleotide and amino acid limitation can drive conflict between hosts and MGEs and that downregulation of host-specific processes like membrane biosynthesis may occur when MGE particles do not require lipids for production.

Finally, we evaluated our intermediary state by setting lower bounds on MGE production for the F128+ model (lower flux bound on plasmid pseudoreaction = 0.9 mmol gDW^−1^ hr^−1^, lower flux bound on phage pseudoreaction = 0 mmol gDW^−1^ hr^−1^) and the F128+ M13+ model (lower flux bound on plasmid pseudoreaction = 0.9 mmol gDW^−1^ hr^−1^, lower flux bound on phage pseudoreaction = 0.06 mmol gDW^−1^ hr^−1^) (Fig. 1; Fig. S1; Table S1). These bounds were chosen because they allowed some amount of bacterial growth in all monoculture and co-culture conditions when running simulations in COMETS using dFBA (Fig. S1). These bounds also matched the lower bound on plasmid production used for F128+ M13+ models in our pFBA and FVA analyses. With these intermediary models, we examined how *E. coli ∆metB* with different carriage statuses was predicted to use and produce different compounds in monoculture. We did so by considering the biomass-normalized concentration (mmol gDW^−1^) of various media metabolites that accumulated during cellular growth due to excretion by *E. coli ∆metB*. We found that wild-type *E. coli ∆metB* produced various carbon waste products at lower concentrations per cell than bacteria carrying MGEs (Fig. 1G; Table S1). dFBA simulations also predicted that the reusable waste products acetate, glycolate, and formate, generated during overflow metabolism, were rapidly reassimilated by wild-type *E. coli ∆metB*, while F128+ and F128+ M13+ *E. coli ∆metB* did not take these products back up at the same rate as wild-type cells (Fig. 1G; Table S1). Because acetate is an organic acid that can be used as a sole carbon source by a wide variety of microbes (57), we expected that its increased accumulation during the growth of *E. coli ∆metB* carrying different MGEs could have significant implications for microbial communities. Carriage also increased the per-cell production of hexanoate and altered the production of ethanol and carbon dioxide (Fig. 1G; Table S1). Altogether, our genome-scale metabolic modeling results predicted that carriage of plasmid-F128 and phage-M13 should alter host metabolism and drive changes in chemical exchange.

### MGE carriage changes *E. coli* growth rate and excretion profile *in vitro*

**Fig 2:**
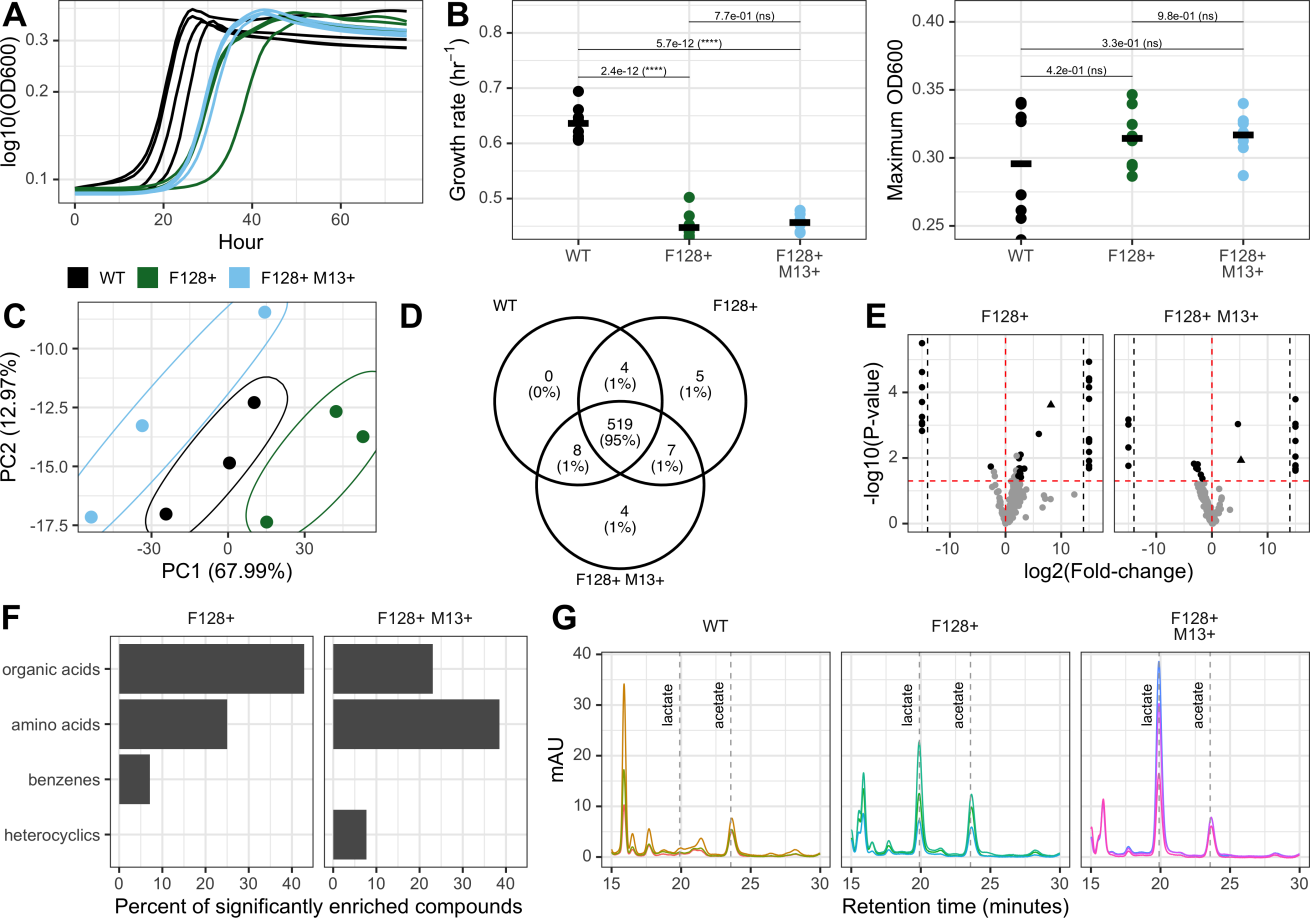
Mobile genetic element carriage by *E. coli* changes host growth rate and excretion profile. (**A**) Log10-transformed OD_600_ growth curves for *E. coli ∆metB* grown in monoculture. Curves are shown for a single experimental run with four biological replicates per condition. (**B**) Estimated log-linear growth rates and maximum measured OD_600_ for *E. coli ∆metB* grown in monoculture. Statistical significance was determined using a one-way ANOVA with Tukey’s honestly significant difference (HSD) test. Data are taken from two independent experiments run during different weeks with four biological replicates per condition per experiment. (**C**) Principal components analysis plot of 548 metabolic features for three *E. coli ∆metB* spent media conditions, each with three biological replicates, including 95% confidence ellipses. (**D**) Venn diagram of the number of metabolites detected in each of three spent media conditions across three biological replicates. (**E**) Volcano plot of targeted metabolites from each type of MGE-positive *E. coli ∆metB* spent media compared to wild-type spent media. Black dots indicate significant compounds with both a VIP >1 and a raw *P* value < 0.05. The triangle corresponds to lactate. Points past the black dotted lines correspond to compounds with infinite log2(fold-change) values. (**F**) Percent of all metabolites significantly enriched (VIP >1, raw *P* value < 0.05, log2(fold-change) >0) in MGE-positive *E. coli ∆metB* spent media compared to wild-type spent media within each compound class. The top three most represented compound classes for each comparison group are shown. (**G**): High-performance liquid chromatography traces for three biological replicates of spent media from *E. coli ∆metB* with various carriage statuses. Retention times for lactate and acetate are labeled with dotted lines and text.

Because metabolic modeling suggested that MGE carriage should change the growth rate, total yield, and effective excretion profile of *E. coli ∆metB* (Fig. 1G; Table S1), we next sought to test these predictions using our *in vitro* system. We transmitted our MGEs of interest to *E. coli ∆metB* and grew these strains in monoculture (lactose and methionine minimal hypho media) to identify potential changes in growth rate or yield (Fig. 2A and B). We found that, consistent with our dFBA modeling predictions (Fig. S1B), carriage reduced the growth rate of hosts relative to wild-type *E. coli ∆metB* (Fig. 2A and B, one-way ANOVA with Tukey’s honestly significant difference [HSD] test, *P* < 5.8e-12), though carrying both MGEs did not change growth rate relative to plasmid carriage alone (Fig. 2A and B, one-way ANOVA with Tukey’s HSD test, *P* = 0.77). Growth rates were calculated with OD_600_ values using a log-linear approach, where the slope of an exponential line fit to the log phase of the growth curve was evaluated as the growth rate (58). In contrast to dFBA (Fig. S1B and D), we observed no change in the productivity (maximum OD_600_) of conditions carrying MGEs (Fig. 2B, one-way ANOVA with Tukey’s HSD test, *P* > 0.33), suggesting a minimal relationship between growth rate and yield, as might be expected under a high efficiency growth strategy (59).

We next sought to understand whether carriage changed the effective excretion profile of *E. coli ∆metB*, as dFBA predicted (Fig. 1G). We grew *E. coli ∆metB* with different carriage statuses to mid-log in monoculture (lactose and methionine minimal hypho media), and Metware Biotechnology Inc. performed ultra-performance liquid chromatography mass spectrometry (LC-MS) on the filtered spent media using their widely targeted metabolomics panel. Mid-log was chosen to capture the secretion of carbon byproducts prior to reuptake and optimize the level of phage infection, given that the number of phage-free cells was expected to increase as cultures approached stationary (40). There were 548 total compounds above the limit of detection in at least one of our three *E. coli ∆metB* conditions. We completed principal components analysis on the subset of *E. coli ∆metB* carriage statuses and found that our three carriage statuses were distinguishable, although there was greater replicate-to-replicate variability in F128+ M13+ spent media and 95% confidence ellipses suggested the potential for overlap between wild-type and F128+ M13+ metabolic profiles (Fig. 2C). There was also significant conservation in the identities of metabolites above the limit of detection across all three conditions. Out of 548 compounds, 519 were detected in all three conditions, with five compounds detected only in F128+ spent media and four compounds detected only in F128+ M13+ spent media (Fig. 2D; Table S1). Seven compounds were detected in both types of MGE-positive spent media but not wild-type spent media (Fig. 2D; Table S2). Given that few compounds were above the limit of detection only in phage-infected conditions, these metabolomic results were not broadly supportive of alternative hypotheses for changes to excretion profile, such as the possibility that phage freed a wide diversity of nutrients via cellular lysis (12) or by increasing general cellular leakiness (60–64). Instead, metabolomics suggested that differences in the excreted compounds were more likely to be the result of changes in the activity of specific metabolic pathways.

To identify potential differences in externalized compounds, we sought to identify differential metabolites in MGE-positive spent media types relative to wild-type spent media. For both relevant pairwise comparisons, we combined the multivariate procedure orthogonal least squares discriminant analysis (OPLS-DA) with univariate student’s t-tests, such that compounds with a variable importance in prediction (VIP) value >1 and a raw *P* value < 0.05 were considered significant (Fig. 2E). We also performed Benjamini-Hochberg correction for multiple comparisons on univariate *P* values (Table S3). Finally, we compared the log2(fold-change) in intensity of each metabolite between relevant conditions. Approximately 10% of the 549 detected compounds (53 total compounds) were identified as potential differential metabolites (VIP >1 and raw *P* value < 0.05) across conditions. Thirty-seven compounds had significantly different concentrations in F128+ spent media relative to wild-type spent media (28 compounds enriched and 9 depleted), while 24 compounds had significantly different concentrations in F128+ M13+ spent media relative to wild-type spent media (13 enriched and 11 depleted) (Fig. 2E; Table S3). Eight compounds were significantly enriched in both MGE-positive conditions, and no compounds were significantly depleted in both MGE-positive conditions (Fig. 2F; Table S3). Most of the compounds that were enriched in MGE-positive spent media were organic or amino acids (Fig. 2F), two types of metabolites likely to be important for supporting the growth of other microbial species and therefore consequential for changing community composition and function. We were particularly interested in the large log2(fold-change) in lactic acid between wild-type and both MGE-positive spent media conditions (Fig. 2E) as it is an organic acid that can be used as a sole carbon source by the two other species in our synthetic system, *S. enterica* and *M. extorquens* (65–67), though it was not significantly enriched (Table S3, adjusted *P* value = 0.59) in F128+ M13+ spent media following adjustment for multiple comparisons.

We validated the enrichment of lactate in MGE-positive spent media by performing high-performance liquid chromatography (HPLC) on new *E. coli ∆metB* monoculture (lactose and methionine minimal hypho media) mid-log spent media samples (Fig. 2G). Following biomass normalization as CFUs at the time of filter sterilization, we found that there was no difference in the amount of acetate produced by all three types of *E. coli ∆metB* (Fig. 2G; Table S4, one-way ANOVA with Tukey’s HSD test, *P* > 0.05), while lactate accumulation only occurred when *E. coli ∆metB* carried MGEs and at similar concentrations regardless of MGE carriage status (Fig. 2G; Table S4, Welch’s *t*-test, *P* = 0.245). Because there were no gene products on plasmid-F128 or phage-M13 that we expected to directly induce the production of lactate, the accumulation of lactate in our MGE-positive conditions instead suggested that the demands of MGE carriage shifted host metabolism to drive lactate production.

### MGE carriage in *E. coli* alters the composition and function of mutualistic bacterial communities

**Fig 3:**
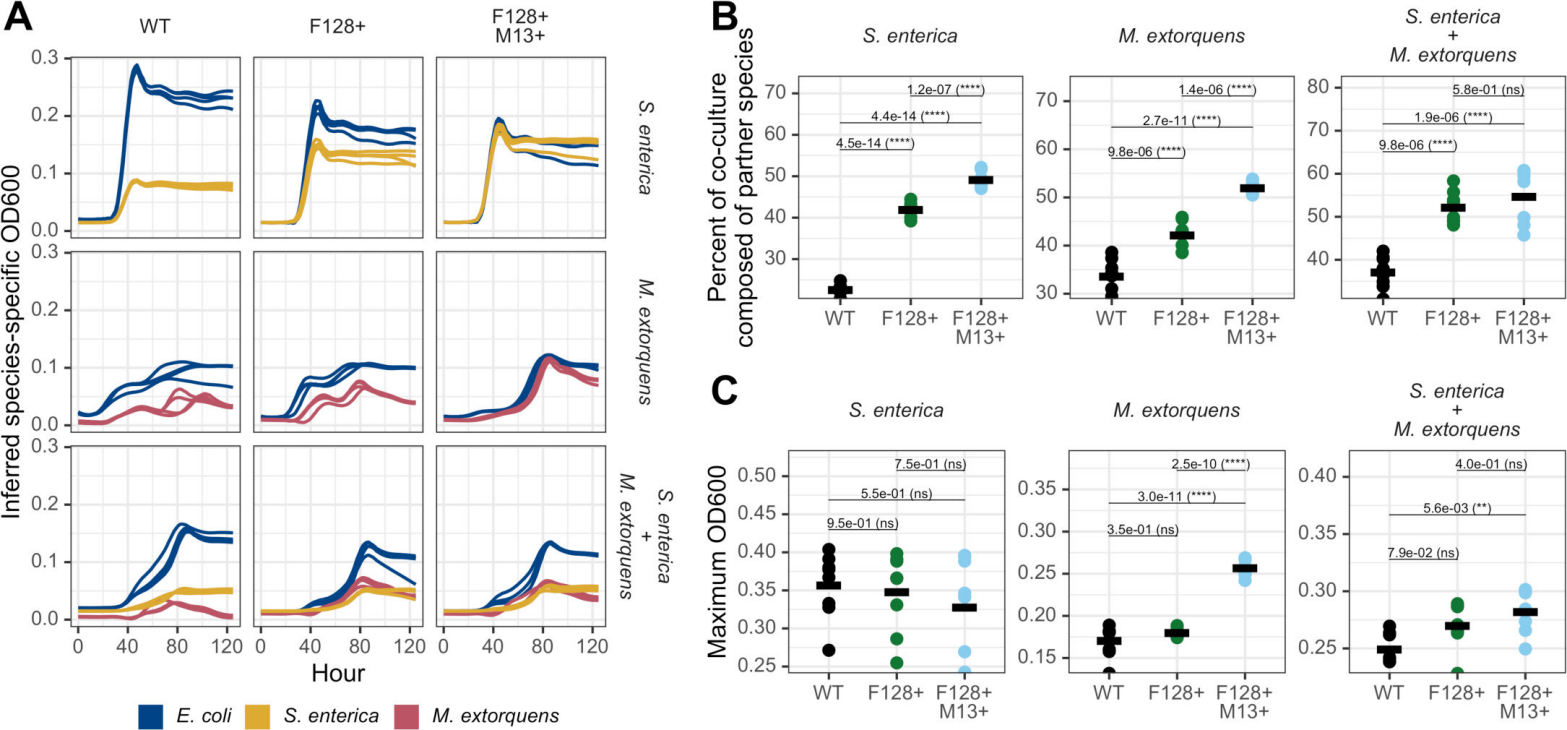
Mobile genetic element carriage by *E. coli* changes the composition and productivity of microbial co-cultures. (**A**) OD_600_ growth curves for two- and three-species cross-feeding bacterial communities grown with *E. coli ∆metB* with different carriage statuses. Inferred species-specific OD_600_ curves were derived from fluorescence values. Curves shown represent a single experimental run with four biological replicates per condition. (**B**) Percent of cross-feeding co-culture composed of partner species, calculated using species-specific OD_600_ values derived from the timepoint with the maximum measured OD_600_. Statistical significance was determined using one-way ANOVA with Tukey’s honestly significant difference (HSD) test. Data were taken from two independent experiments run during different weeks with four biological replicates per condition per experiment. (**C**) Maximum total OD_600_ of two- and three-species cross-feeding bacterial communities. Statistical significance was determined using one-way ANOVA with Tukey’s HSD test. Data were taken from two independent experiments run during different weeks with four biological replicates per condition per experiment.

Because we observed a reduction in growth rate and an increased accumulation of organic acids due to carriage *in vitro*, we hypothesized that in our two- and three-species bacterial obligate mutualisms, MGE carriage should increase the density of partner species. We tested this hypothesis by performing co-culture experiments using our synthetic mutualisms (Fig. 1A). Cross-feeding co-cultures were grown in each of three appropriate nutrient conditions: (i) the *E. coli ∆metB* and *S. enterica* mutualism was grown in lactose minimal hypho media, where *E. coli ∆metB* provides carbon and *S. enterica* provides methionine; (ii) the *E. coli ∆metB* and *M. extorquens* mutualism was grown in lactose, methylamine, and methionine minimal hypho media, where *E. coli ∆metB* provides carbon, uses exogenous methionine, and *M. extorquens* metabolizes methylamine to nitrogen that is usable for *E. coli ∆metB*; and (iii) the tripartite mutualism between *E. coli ∆metB*, *S. enterica,* and *M. extorquens* was grown in lactose and methylamine minimal hypho media, where *E. coli ∆metB* provides carbon, *S. enterica* provides methionine, and *M. extorquens* metabolizes methylamine to nitrogen that is usable for *E. coli ∆metB* and *S. enterica* (Fig. 1A). We found that carriage increased partner density regardless of partner identity or community complexity (Fig. 3A). This result was broadly consistent with dFBA predictions of species ratios in mutualistic bacterial communities given MGE carriage in *E. coli ∆metB* (Fig. S1B and C). In addition, in all cases except the three-species condition (Fig. 3B, one-way ANOVA with Tukey’s HSD test, *P* = 0.58), *in vitro* carriage of both the plasmid and phage increased bacterial partner density over carriage of the plasmid alone (Fig. 3B, one-way ANOVA with Tukey’s HSD test, *P <* 1.5e-6). Carriage also increased the productivity (maximum OD_600_) of the whole bacterial community in some co-cultures. In the two-species co-culture with *M. extorquens,* carriage of both MGEs increased community productivity relative to co-cultures with either wild-type and F128+ *E. coli ∆metB* (Fig. 3C, one-way ANOVA with Tukey’s HSD test, *P <* 2.6e-10). Carriage of both MGEs also increased productivity in the three-species co-culture relative to wild-type *E. coli ∆metB* (Fig. 3C, one-way ANOVA with Tukey’s HSD test, *P* = 0.0056). These results demonstrated that sustained MGE carriage in a single bacterial species was sufficient to significantly alter microbial community composition (as the relative abundance of bacterial species) and function (as maximum OD_600_) via host metabolic remodeling.

### In a synthetic obligate mutualism, partner species use a combination of metabolites from *E. coli* to support growth

**Fig 4:**
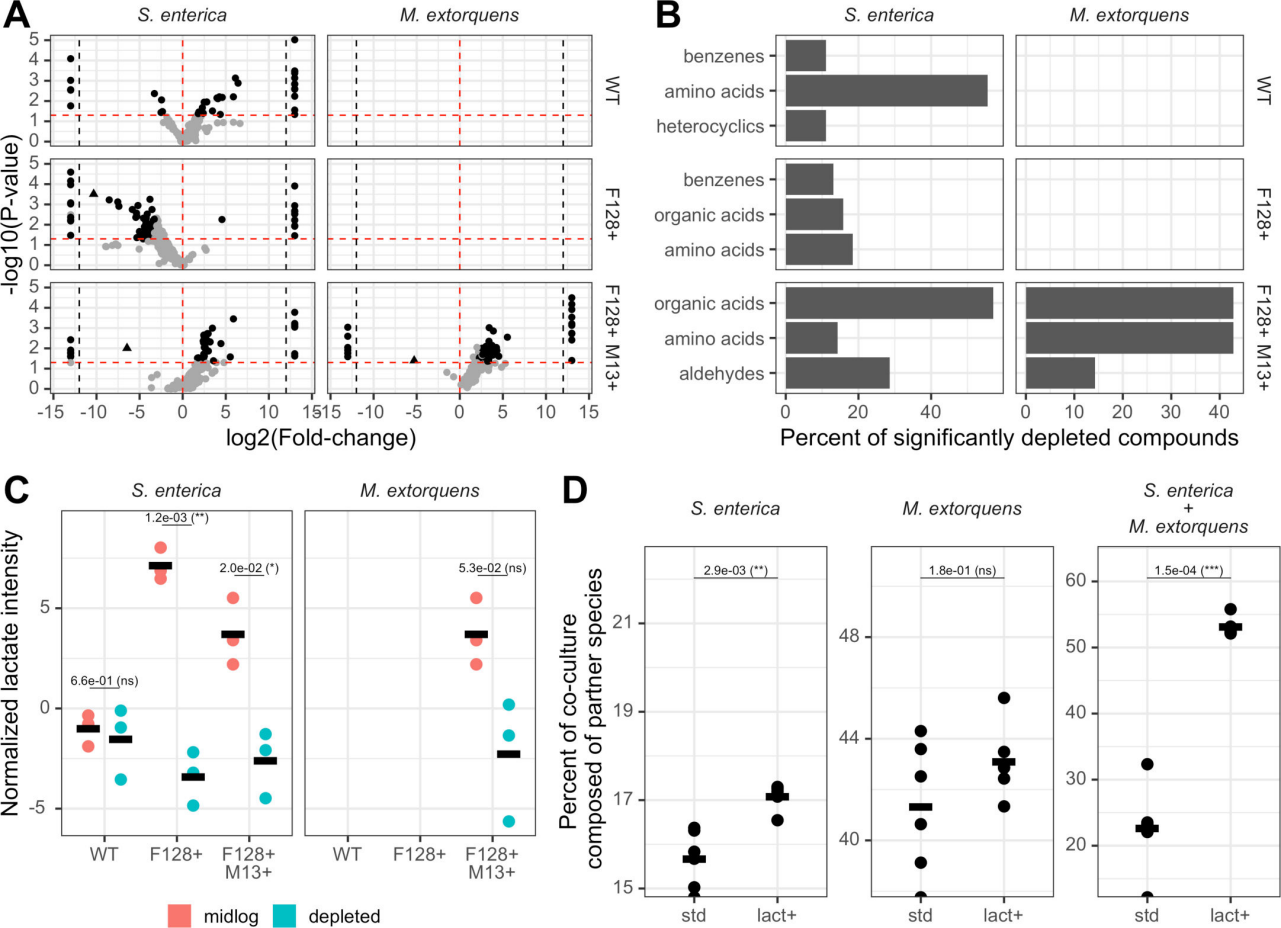
Partners use a combination of metabolites from *E. coli ∆metB* to support growth in mutualism. (**A**) Volcano plot of targeted metabolites from both types of partner species grown in each type of *E. coli ∆metB* spent media. Black dots indicate significant compounds with both a VIP >1 and a raw *P* value < 0.05. The triangle corresponds to lactate. Points past the black dotted lines correspond to compounds with infinite log2(fold-change) values. No data are available for *M. extorquens* grown in wild-type or F128+ spent media. (**B**) Percent of all metabolites significantly depleted (VIP >1, raw *P* value < 0.05, log2(fold-change) <0) by partner species from *E. coli ∆metB* spent media within each compound class. The top three most represented compound classes for each comparison group are shown. No data are available for *M. extorquens* grown in wild-type or F128+ spent media. (**C**) Normalized lactate intensity between mid-log *E. coli ∆metB* spent media and partner-depleted spent media. Statistical significance was determined using student’s t-tests with Benjamini-Hochberg correction for multiple comparisons. No data are available for *M. extorquens* grown in wild-type or F128+ spent media. Three biological replicates were completed for each condition. (**D**) Percent of final co-culture composed of partner species during growth in obligate mutualism with wild-type *E. coli ∆metB* with or without 1 mmol lactate supplemented into the cross-feeding media, calculated using species-specific OD_600_ values derived from the timepoint with the maximum measured OD_600_. Statistical significance was determined using Welch’s t-tests with Benjamini-Hochberg correction for multiple comparisons. Data are taken from one independent experiment with six biological replicates per condition.

Next, we attempted to determine the metabolic basis of changes in bacterial community composition. We considered what compounds were depleted by partner species from the spent media of *E. coli ∆metB* with different carriage statuses. To do so, we grew our partner species *S. enterica* and *M. extorquens* to stationary phase in mid-log spent media from our three *E. coli ∆metB* carriage statuses, and performed LC-MS using a widely targeted metabolomics panel on the filtered stationary spent media. In this spent media, the only carbon sources available to *S. enterica* and *M. extorquens* were compounds externalized by *E. coli ∆metB* during growth on lactose and methionine. As previously described, we used OPLS-DA and student’s t-tests to identify potential differential metabolites between *E. coli ∆metB* mid-log spent media and the corresponding partner-depleted conditions, such that compounds with a VIP value >1 and a raw *P* value < 0.05 were considered significant (Fig. 4A; Table S5). As before, we also performed Benjamini-Hochberg correction for multiple comparisons on univariate *P* values and calculated log2(fold changes) in metabolite intensity (Table S5). Using the cut-offs of VIP >1 and raw *P* value < 0.05, we found that, of these potential differential metabolites, 9 compounds were depleted by *S. enterica* from wild-type media, 76 compounds were depleted by *S. enterica* from F128+ media, and 7 compounds were depleted by both *S. enterica* and *M. extorquens* from F128+ M13+ media (Fig. 4A; Table S5). Most of these compounds could not be used by either partner as a sole carbon source, indicating that their depletion likely could not support partner growth without the presence of other known system-specific compounds like acetate, galactose, and several B vitamins (68). Glyceraldehyde—a compound detected in F128+ and F128+ M13+ spent media but below the limit of detection in wild-type spent media and an intermediate in glucose/fructose/galactose/lactose metabolism (69)—was not detected in any MGE-positive spent media conditions following the growth of both partners (Fig. 4A and B; Table S5). Similarly, two organic acid derivatives, chlorogenic acid and 3-carboxypropyltrimethylammonium, were depleted by both partner species from F128+ M13+ spent media (Fig. 4A and B; Table S5). Altogether, our metabolomics results suggested that several compounds could be important for supporting partner growth and that key compounds likely varied between our two partner species, a finding that was broadly supported by dFBA (Fig. S1E and F).

Given the observed accumulation of lactate in our MGE-positive conditions, and the fact that it is one of the few compounds in our targeted metabolomics panel that could be used as a sole carbon source by both *S. enterica* and *M. extorquens* (65–67), we investigated its depletion in our metabolomics data. We found that lactate was significantly depleted by *S. enterica* from F128+ and F128+ M13+ media (Fig. 4C, student’s t-test with Benjamini-Hochberg correction, *P* < 0.02). There was also a large decrease in lactate from F128+ M13+ spent media following the growth of *M. extorquens*, although this change was not statistically significant following correction for multiple comparisons (Fig. 4C, student’s t-test with Benjamini-Hochberg correction, *P* = 0.053). Using the more conservative threshold of correction for multiple comparisons against all metabolites, only the depletion of lactate by *S. enterica* from F128+ spent media was significant (Fig. S5). Regardless, the large log2(fold-change) in lactate between MGE-positive spent media and partner-depleted spent media broadly supported our expectation that lactate production by MGE-positive *E. coli ∆metB* could be one driver of the increased partner density and total productivity observed in our synthetic microbial communities. To quantify the expected contribution of lactate to community composition, we grew our two- and three-species cross-feeding bacterial communities with wild-type *E. coli ∆metB* and supplemented with 1 mmol lactate, which corresponded to the approximate expected co-culture concentration of secreted lactate during MGE carriage based on our HPLC results (Fig. 2G). We found that the addition of lactate increased *S. enterica* density in bipartite growth (Fig. 4D*,* Welch’s t-test,* P* = 0.0029); however, the magnitude of this difference did not match the observed compositional differences when *E. coli ∆metB* was carrying our MGEs of interest (Fig. 3B, >40% *S. enterica*). In addition, though lactate supplementation did not change *M. extorquens* density during bipartite growth (Fig. 4D, Welch’s t-test with Benjamini-Hochberg correction,* P* = 0.18), total partner density in tripartite mutualism did increase with lactate addition (Fig. 4D, Welch’s t-test with Benjamini-Hochberg correction,* P* = 1.5e-4) and roughly matched the partner density observed when both partners were co-cultured with *E. coli ∆metB* F128 +M13+ (Fig. 3B, ~55% partner species). While there are multiple reasons that exogenous lactate addition could have different effects on bacterial community composition across conditions in comparison to increased lactate secretion (70–72), most parsimoniously, these results suggested that lactate secretion alone was not sufficient to explain the changes in species ratios that we observed in our community composition experiments.

### Changes in bacterial species densities during mutualistic growth with MGE-positive *E. coli* are reproducible across bacterial interactions and *E. coli* genotype

**Fig 5:**
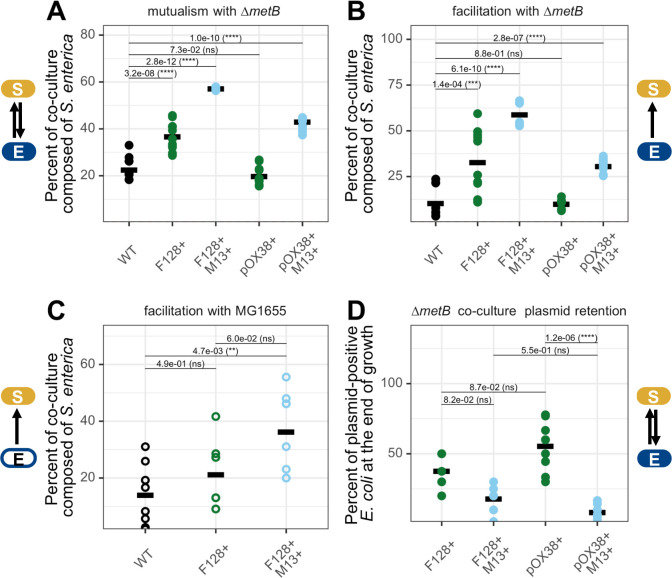
Changes in partner density occur across host genotypes, bacterial interactions, and mobile genetic elements. (**A**) Percent of maximum co-culture density composed of *S. enterica* when growing in obligate mutualism with *E. coli ∆metB* across carriage statuses. Statistical significance was determined with Welch’s t-tests using “WT” as a reference group and Benjamini-Hochberg correction for multiple comparisons. Data were taken from three independent experiments run during different weeks with three biological replicates per condition per experiment. Percentages were calculated using species-specific OD_600_ values derived from the timepoint with the maximum measured OD_600_. (**B**) Percent of maximum co-culture density composed of *S. enterica* when growing in unidirectional facilitation with *E. coli ∆metB* across carriage statuses. Statistical significance was determined with Welch’s t-tests using “WT” as a reference group and Benjamini-Hochberg correction for multiple comparisons. Data were taken from three independent experiments run during different weeks with three biological replicates per condition per experiment. Percentages were calculated using species-specific OD_600_ values derived from the timepoint with the maximum measured OD_600_. (**C**) Percent of maximum co-culture density composed of *S. enterica* when growing in unidirectional facilitation with *E. coli* MG1655 (open circles) across carriage statuses. Statistical significance was determined using a one-way ANOVA with Tukey’s honestly significant difference (HSD) test. Data were taken from two independent experiments run during different weeks with four biological replicates per condition per experiment. Percentages were calculated using CFUs/mL of each strain at the end of growth as plated on appropriate selective agar media. (**D**) Percent of final *E. coli ∆metB* population still plasmid-positive in stationary phase following growth in obligate mutualism with *S. enterica*. Percentages were calculated using CFUs/mL of each strain at the end of growth as plated on appropriate selective agar media. Statistical significance was determined using one-way ANOVA with Tukey’s HSD test. Data were taken from three independent experiments run during different weeks with three biological replicates per condition per experiment.

Finally, we investigated the generality of our experimental observation that MGE carriage can change species ratios during mutualistic bacterial growth. To do so, we obtained an additional, condensed version of the conjugative F-plasmid known as pOX38 (73). pOX38 is a ~ 60 kb plasmid that primarily encodes the conjugative machinery and two types of toxin-antitoxin system (73) (Table S6), in contrast to plasmid-F128, which is much larger at ~230 kb and includes multiple insertion sequence (IS) elements, three classes of toxin-antitoxin systems, and the lac operon in addition to the conjugative machinery (Table S7 and S8). Because we observed the largest magnitude change in relative species ratios in the two-species cross-feeding communities consisting of *E. coli ∆metB* and *S. enterica* in our initial community composition experiments (Fig. 3A and B, ~30% increase in percent partner versus ~20%)*,* we chose to test only this condition in downstream experiments. We grew *E. coli ∆metB* carrying pOX38 or both pOX38 and the phage-M13 under conditions of obligate mutualism with *S. enterica* (lactose minimal hypho media) (Fig. 5A). We found that carrying pOX38 alone did not change the density of *S. enterica* relative to growth with wild-type *E. coli* (Fig. 5A, Welch’s t-test with Benjamini-Hochberg correction, *P* = 0.073), contrary to our previous finding that the carriage of plasmid-F128 increased *S. enterica* density during co-culture growth. By contrast, the carriage of both pOX38 and phage-M13 increased *S. enterica* density as previously observed in conditions with both plasmid-F128 and phage-M13 (Fig. 5A, Welch’s t-test with Benjamini-Hochberg correction, *P* = 1.0e-10). The divergence between bacterial species ratios during growth with F128+ versus pOX38+ suggested that the metabolic burden of pOX38 could be less than that of plasmid-F128 (Fig. 5A). This finding was supported by the growth of these strains in monoculture (lactose and methionine minimal hypho media), where pOX38 carriage resulted in a much smaller reduction in growth rate (Table S9, difference of 0.045 hr^−1^) relative to wild-type* E. coli ∆metB* than carriage of plasmid-F128 (Fig. 2B, difference of 0.188 hr^−1^). However, the use of different plasmid genotypes also underscored that phage-M13 drove differences in bacterial species ratios regardless of the version of the conjugative plasmid it used for cellular entry, suggesting reproducibility in the metabolic conflict imposed by the phage on the *E. coli ∆metB* background (Fig. 5A and B).

Next, we tested the impact of MGE carriage on engagement in different ecological interactions between bacteria by growing *E. coli ∆metB* with *S. enterica* under facilitative nutrient conditions (lactose and methionine minimal hypho media) and observing the final species ratios of the bacterial community (Fig. 5B). During facilitation, *E. coli ∆metB* is freed from metabolic dependence on *S. enterica* due to the addition of methionine into the medium, while *S. enterica* remains dependent on *E. coli ∆metB* for carbon. This unidirectional dependence typically results in a community that is highly biased toward *E. coli ∆metB,* and the nutrient conditions provide a metabolically congruent environment to monoculture for *E. coli ∆metB*, as the strain is never under conditions of methionine limitation. We found that, consistent with our results in obligate mutualism, bacterial species ratios changed during carriage of plasmid-F128, with increased partner density occurring during growth with MGE-positive *E. coli ∆metB* (Fig. 5B, Welch’s t-test with Benjamini-Hochberg correction, *P* = 1.4e-4). We also observed that, as in the case of obligate mutualism, carriage of pOX38 alone did not change bacterial species ratios relative to wild-type *E. coli ∆metB* (Fig. 5B, Welch’s t-test with Benjamini-Hochberg correction, *P* = 0.88), while carriage of both pOX38 and phage-M13 did (Fig. 5B*,* Welch’s t-test with Benjamini-Hochberg correction, *P* = 2.8e-7). Taken together, we found that phage carriage increased partner density regardless of host interactions, and, while individual conjugative plasmids had reproducible effects between the two types of bacterial interactions, different plasmid genotypes had different overall effects on bacterial species ratios (Fig. 5B).

While our results suggested that changes to bacterial species ratios were not generalizable across different plasmid genotypes, we also tested whether MGE carriage increased partner density regardless of the genetic background of the host. We expected that the host genetic background should matter because it could alter the metabolic pathways accessible to the host and change the complementarity between the intracellular resources required by a host versus its MGEs. We tested this expectation by transmitting plasmid-F128 or plasmid-F128 and phage-M13 to a prototrophic *E. coli* MG1655 strain with the lac operon. We grew these strains in lactose minimal hypho media with *S. enterica* to replicate the nutrient conditions of facilitation with *E. coli ∆metB*, because MG1655 can provide carbon byproducts to *S. enterica* without depending on *S. enterica* for any nutrients. These results broadly matched our facilitative findings with *E. coli ∆metB*, such that carriage of both MGEs increased the density of *S. enterica* (Fig. 5C, one-way ANOVA with Tukey’s HSD test, *P* = 0.0047). However, unlike facilitative growth with *E. coli ∆metB*, during facilitative growth with MG1655, *S. enterica* never dominated the population regardless of partner carriage status (Fig. 5C). This suggested that while shifts in bacterial species ratios can be qualitatively reproducible across host genetic backgrounds, host identity likely matters for the precise metabolic manifestations of MGE carriage and the extent of compositional changes in a bacterial community.

Finally, we tested the retention rates of our MGEs during *E. coli ∆metB*’s growth in obligate mutualism with *S. enterica* by quantifying the number of plasmid-positive cells at the end of growth. We were interested in how quickly *E. coli ∆metB* cells lost our MGEs of interest via cellular division or the emergence of resistance, since metabolic conflict should only occur in the fraction of the population carrying MGEs. We expected that rates of loss could differ between monoculture and co-culture due to differences in growth rate and the relative metabolic burden of carriage; we focused on the rate of loss during obligate mutualism because we anticipated it should be more informative about the consequences of carriage for the multispecies microbial communities we sought to consider. We found that infection with phage-M13 increased the rates of pOX38 loss relative to carriage of pOX38 alone (Fig. 5D, one-way ANOVA with Tukey’s HSD test, *P* = 1.2e-6). In addition, rates of plasmid loss were high across all conditions (Fig. 5D), with at least ~45% of the *E. coli ∆metB* population plasmid-free at the end of growth regardless of plasmid type or phage carriage. This result indicated that *E. coli ∆metB* populations were heterogeneous and, in many cases, dominated by parasite-free cells at the end of growth, suggesting that we were underestimating the true metabolic burden of all three MGEs and the changes in bacterial species ratios they could drive because we were not able to grow strains under antibiotic selection in co-culture. Taken together, our results supported previous work suggesting that diverse MGEs can cause highly specific remodeling of their hosts that varies with host identity and depends additionally upon MGE genomic content (74), the complementarity in amino acid and nucleotide composition between MGE and host (10), MGE host range, and MGE lifecycle (11). Importantly, our results also empirically demonstrated that MGEs can reshape microbial communities by changing host growth rate and metabolite exchange, regardless of the precise metabolic changes that MGEs induce in their hosts.

## DISCUSSION

Our study examined how metabolic conflict between *E. coli* and two common MGEs impacted microbial community composition and function in the absence of well-explored, direct mechanisms like lysis or horizontal gene transfer. We integrated a genome-scale metabolic modeling approach with *in vitro* experiments to address this question. In our modeling, we used the nucleotide and amino acid content of two MGEs to impose a metabolic cost on the host cell, in alignment with previously developed methods to evaluate the metabolic consequences of parasite infection (51–54). Results from these models predicted that MGE carriage could change demand for amino acids, reduce host growth rate, and alter metabolite excretion profiles. Our *in vitro* experiments confirmed that carriage altered host growth rate and excretion profile, in particular driving the externalization of lactate. Importantly, *in vitro* experiments clearly demonstrated that the metabolic effects of carriage on one host can significantly alter the composition and function of multispecies microbial communities. Our work, therefore, suggests that while the genetic background of both hosts and MGEs can contribute to the metabolic burden of MGE carriage and therefore the specific phenotypic manifestations of metabolic conflict (75), the indirect metabolic consequences of carriage for host cells are sufficient in some cases to change host contributions to community function.

Our FBA results first predicted that the carriage of plasmid-F128 and phage-M13 should drive remodeling of *E. coli ∆metB* metabolism. Because MGE biomass reactions were composed of nucleotides and amino acids only, we found that forcing MGE production increased amino acid biosynthesis and downregulated host-specific lipid pathways. Our modeling results aligned with existing metabolic approaches to modeling parasitic infection. These models have previously been used to demonstrate that lytic phage infection reduces flux toward cell wall, lipid, and cofactor metabolism (52, 53). In addition, modeling the RNA phage MS2 in an *E. coli* metabolic system showed that infection upregulates the pentose phosphate pathway and the biosynthesis of most amino acids while downregulating the citric acid cycle, findings which we also observed in our system despite the mechanistic and genetic differences of plasmid-F128 and phage-M13 compared to MS2 (53). Finally, we found that our modeling approach predicted that MGE carriage in *E. coli ∆metB* should change host excretion profile by increasing the concentration of key organic acid byproducts like acetate produced per cell while reducing the ability of these cells to take up these byproducts to support additional growth.

While our modeling aligned well with existing work and predicted several key host changes *in vitro*, there are caveats to our *in silico* findings. Most obviously, FBA did not predict the observed accumulation of lactate that occurred because of MGE carriage in our *in vitro* system. We expect that our modeling predictions could be improved by imposing more biologically realistic proteomic constraints. For example, **C**onstrained **A**llocation **F**lux **B**alance **A**nalysis (CAFBA) (76) and variants of **F**lux **B**alance **A**nalysis **w**ith **M**olecular **C**rowding (FBAwMC) (77) have been demonstrated to better reflect shifts between fermentation and respiration processes and the accumulation of resultant waste products (76, 77). Alternatively, the divergence between *in silico* and *in vitro* results could be due to a lack of metabolic optimality exhibited by MGE-positive cells (a core assumption of FBA), given that *in vitro* MGE carriage was novel and we did not allow time for coevolution. There is room for extensive future work to improve predictions of the metabolic impacts of novel MGE carriage.

In alignment with our modeling results, our *in vitro* study also suggested that carriage of our MGEs of interest reshaped *E. coli ∆metB* metabolism through changes to both growth rate and excretion profile. These changes had consequences for the composition and function of our two- and three-species cross-feeding bacterial communities regardless of partner identity or community complexity. In addition, we found that MGE carriage drove the accumulation of lactate, an organic acid with potential consequences for supporting higher densities of partner species in our synthetic microbial communities, though our results do not suggest that the induction of lactate alone was sufficient to drive the changes we observed in community composition. Instead, we expect that the shifts in bacterial species ratios are due to the combined effects of reduced host growth rate and changes to excretion profile that extend beyond lactate production. Nevertheless, if lactate accumulation occurs at appreciable levels in natural communities because of filamentous phage or conjugative plasmid carriage and is a reproducible phenotype across hosts and MGE pairs, this could be especially important for the design and management of a wide range of microbiomes. Organic acids produced by *E. coli* during overflow metabolism can suppress the growth of species like *Pseudomonas aeruginosa* (78), and lactate, due to its acidity, can be a particularly inhibitory metabolite at high concentrations and low pH (65). In human-associated microbiomes, lactate can also promote the growth of unwanted pathogens like *S. enterica* and *Candida albicans* (79, 80). Our results emphasize that the metabolic remodeling of bacterial hosts by MGEs likely serves as an important contributor to community structure and function, with consequences that may cascade beyond the microbial communities in which they are found.

However, though we observed that *E. coli ∆metB* cells carrying our MGEs of interest produced lactate, we could not identify the mechanistic basis of this phenotype. There are several possibilities that deserve further investigation. For one, changes in bacterial excretion profile have been linked to changes in the ratio of cell surface area to volume (S/V) (81–83), such that an increased S/V ratio can cause increased cellular leakiness (81) and alter the dominance of respiration versus fermentation metabolic processes in *E. coli* (82). If the carriage of certain MGEs can contribute to alterations in the cell S/V ratio, such changes could drive differential production of lactate and other organic acids relevant to our cross-feeding system. A related possibility is that the production of the F128-associated conjugative pilus and phage-M13 extrusion channels (84, 85) could push cells toward fermentative processes by reducing the enzymatic capacity of the inner membrane (81). There is also the possibility that lactate production is related to the induction of the phage-shock protein (Psp), which, while it was originally discovered to activate in response to the phage-M13 gene product pIV (86), can also be induced by other stressors that reduce a cell’s energetic status (87) or result in increased inner-membrane permeability (88). However, we expect that the most parsimonious explanation for lactate production is the fact that the proteomic cost of respiration exceeds that of fermentation, particularly in terms of nitrogen investment (89). The metabolic burden of producing nitrogen-rich MGE particles like plasmids and phage may require metabolic strategies that increase proteomic efficiency, driving increased overflow metabolism (89). The extent to which this phenotype is generalizable across MGE-host pairings warrants continued attention.

In addition, our results suggested that different MGE genotypes are likely to drive different types of metabolic remodeling, although we could not identify the exact mechanistic underpinnings of the community differences between plasmid-F128 and pOX38. We expect that a major contributing factor is the fact that these plasmids have varying genomic content. Because transposition of IS elements encoded on conjugative plasmids can mediate bacterial adaptation (90), the presence of IS elements on plasmid-F128 could have promoted gene inactivation in plasmid-positive *E. coli ∆metB *in metabolically consequential ways. Furthermore, toxin-antitoxin systems have been shown to induce metabolic stress (91), which may have contributed to more substantial remodeling of *E. coli ∆metB* by plasmid-F128. The presence of the lac operon on plasmid-F128 also represents a potential explanation. Though the F-plasmid has a host range limited to the Enterobacteriaceae (92), carriage of plasmid-F128 can enable lactose utilization in *S. enterica* (93), meaning that in co-culture with F128+ *E. coli ∆metB*, conjugation between the two strains could have freed a subset of *S. enterica* from nutrient dependence (93, 94). Therefore, the fact that pOX38 does not carry the lac operon could explain the divergence in species ratios between these two MGE conditions. However, we did not observe the restoration of lactose utilization by *S. enterica* grown in co-culture (Fig. S2A) in any of our experiments, likely because cultures were grown in liquid at small volumes. Though conjugation between *S. enterica* LT2 and our F128+ *E. coli* strain was detected at moderate rates on agar plates (Fig. S2B, 5-20%), in liquid, if conjugation occurred, it did so at rates below our limit of detection (Fig. S2C, <1%). As a result, we do not expect that transfer of the lac operon—nor the process of conjugation as a whole—was primarily responsible for the observed changes in community composition and function, though it could be a significant force in natural communities (27, 95–97). Instead, our results emphasize that the exact metabolic manifestations of MGE carriage are likely specific to host-MGE pairs and may need to be evaluated on that basis.

To that end, our results lay the foundation for interesting future work. First, we suggest that it will be important to better understand the community-wide consequences of maintaining multiple MGEs in a single host. For example, while the metabolic cost to hosts often increases with the number of plasmids carried (30, 98), in some cases, positive epistasis between plasmids can reduce the cost of acquisition (99). The extent of metabolic remodeling by an MGE and its community consequences is therefore likely to depend on the other MGEs with which it co-occurs. Understanding how metabolic remodeling changes over evolutionary time is also essential to predicting and managing microbial communities. Long-term associations between MGEs and hosts are often enabled by compensatory mutations that accumulate rapidly to ameliorate the costs of carriage (3, 43, 100). Therefore, it is possible that coevolution between *E. coli* and the MGEs tested here would minimize the extent of metabolic modeling. Because MGE carriage is often transient in natural communities, we expect that our results provide a snapshot of how carriage can alter species coexistence when hosts are confronted with novel MGEs, though we suggest evolutionary studies would be an important next step. Finally, because poor nutrient conditions have been found to maintain filamentous phage infections by increasing the cost of resistance mutations (101), microbial community composition and MGE carriage are almost certain to be engaged in feedback loops across ecosystems, where resource availability driven by cross-feeding and competition change host carriage dynamics and host carriage then alters nutrient concentrations by changing host metabolism.

In this study, we combined a genome-scale metabolic modeling approach with ecological experiments to explore the role of metabolic conflict between hosts and MGEs in shaping community composition and function. Our work demonstrates empirically that metabolic conflict between microbial hosts and MGEs is sufficient to shift the structure of the broader community and underscores the diversity of mechanisms through which MGEs can drive microbial community and ecosystem function. Understanding the higher-order ecological effects of host-MGE interactions is central to our ability to manage human-associated microbial communities and design microbiomes for important purposes like bioremediation. Given the ubiquity of MGE carriage across all environments where microbes are found, future work should focus on identifying patterns in how MGE carriage reshapes microbial community function across host-MGE pairs, MGE-MGE interactions, and diverse ecosystems.

## MATERIALS AND METHODS

### Flux balance analysis

Flux balance analysis has been extensively described. It is a linear optimization method used to predict the behavior of metabolic networks (50). By assuming a steady state, FBA optimizes a set of fluxes given an objective function and bounds on the flux allowed through each reaction (50). The linear optimization is formalized as: 


$$maximize\;Z=c^{T}v$$

$$subject\;to\;Sv=0\;and\;lb_{i}\leq v_{i}\leq ub_{i}$$

where *S* is the stoichiometric matrix for all metabolites across all reactions, *v* is the vector of reaction fluxes, and *c* is the vector specifying the objective function *Z* (50). Constraints on the flux through each reaction *v_i_* are imposed using lower (*lb_i_*) and upper flux (*ub_i_*) bounds (50). Using this formalization, we performed FBA, pFBA, and FVA. For pFBA, in addition to the previously specified constraints, an additional constraint is imposed to minimize total flux through the system (i.e., minimize the sum of vector *v*). FVA provides the range of flux values for each reaction that could satisfy the linear optimization problem as it is formulated above.

All simulations were completed using variants of existing metabolic models. The host *E. coli* metabolism used in this study was based on the model iJO1366 (102). To reproduce our *in vitro* system, a version of *E. coli* that is auxotrophic for methionine was created by knocking out the function of the *metB* gene in iJO1366 (103). A methionine-secreting version of *S. enterica* was built using the STM_v1_0 metabolic network by forcing the model to generate 0.5 mmol of extracellular methionine for each gram of biomass growth and preventing reuptake by making methionine transport unidirectional (103). Finally, an *∆hprA M. extorquens* AM1 metabolic model was constructed as previously described (103). Versions of the *E. coli* metabolic model corresponding to MGE carriage were generated and evaluated as described below.

### Generation of MGE biomass functions

To integrate our MGEs of interest into our metabolic models, we defined a pseudoreaction accounting for the stoichiometry required to produce a single MGE particle. Pseudoreactions were generated based on publicly available genome and protein sequence information for the dsDNA conjugative plasmid F128 (GenBank: NZ_CP014271), augmented with tetracycline-resistance genes from *Tn*10 (GenBank: AY528506) and the ssDNA filamentous phage M13 (GenBank: JX412914). Sequencing was performed on our *in vitro* MGE strains to confirm that their content aligned with the GenBank entries we used for genome-scale metabolic model integration. The handful of polymorphisms identified in our strains relative to the reference strains (Table S10) were incorporated into the genomic and proteomic sequences used for downstream model generation.

In line with protocols developed for the integration of various intracellular parasites into host cell metabolic models (54), we first calculated the total moles of each nucleotide per mole of MGE particle, as appropriate for either dsDNA or ssDNA, such that


$$N_{i}^{tot}=\;C_{g}\left(N_{i}^{G}+N_{i}^{int}\right)$$

where $N_{i}^{G}$is the total number of nucleotide *i* in the MGE genome, $N_{i}^{int}$is the total number of nucleotide *i* present in any relevant replication intermediates, and their sum is multiplied by $C_{g}$, the genome copy number per MGE particle, which for both MGEs was a value of 1. We converted the moles of each nucleotide into grams of nucleotide per mole of MGE by multiplying $N_{i}^{tot}$ by the molar mass (g mol^−1^) of the nucleotide *i.* Lastly, we expressed the nucleotide stoichiometric coefficients in millimoles per gram of MGE by dividing $N_{i}^{tot}$ by the total molar weight of the MGE (including both nucleotides and amino acids) and multiplying that value by 1,000.

The total moles of each amino acid per mole of MGE were calculated such that


$$AA_{j}^{tot}=\;\sum_{j}^{k}C_{k}\left(AA_{j}^{k}\right)$$

where $C_{k}$ is the copy number of protein *k,* and ${AA}_{j}^{k}$ is the total number of amino acid *j* present in protein *k.* For the construction of the plasmid-F128 pseudoreaction, only the 24 proteins associated with pilus formation (Table S11) were assigned a copy number greater than one (104). The copy numbers of pilus-associated proteins were calculated based on experimental findings that the pilus is composed of a 5-start helix in which there are 12.8 units of propilin protein per helical turn (105), with a typical maximum total length of 20 microns (106). This provided a rough estimate for the maximum copy number of *traA*, the propilin component. All other pilus-associated proteins were scaled appropriately from that number based on their function in pilus formation and conjugation (Table S11) (107–114). Amino acid investment in plasmid production could therefore be altered based on the total number of pili produced. For our simulations, we imposed a cost of producing only a single pilus structure. Copy numbers of filamentous phage M13 proteins were obtained based on existing work detailing the structure and function of the 10 proteins encoded in the M13 genome (115–118) (Table S12). We then converted the moles of amino acid per mole of MGE to grams of amino acid per mole of MGE by multiplying total moles of each amino acid per mole of MGE by the molar mass (g mol^−1^) of the appropriate amino acid. Finally, as with nucleotides, we expressed the stoichiometric coefficients of each amino acid in the MGE pseudoreaction in millimoles per gram of MGE using the total molar weight of the MGE.

We accounted for the use of ATP molecules to polymerize amino acid monomers by multiplying the expected number of ATP molecules required per peptide bond (4) (54) with the total amino acid counts, performing the calculation for each relevant protein, scaling the value by the assigned protein copy number, and summing across all proteins to generate the total number of ATP molecules required to produce all proteins associated with an MGE. Again, as before, we expressed the stoichiometric coefficients of ATP in the MGE pseudoreaction in millimoles per gram of MGE using the total molar weight of the MGE. We also accounted for the creation of pyrophosphate (PP_i_) molecules formed via the nucleotide polymerization using the same process, by multiplying the expected PP_i_ generated per nucleotide (1) (54) with the total nucleotide counts. The same calculation was applied to relevant genome intermediates, and the results were summed to generate the total number of PP_i_ molecules produced during genome polymerization for an MGE. We expressed the stoichiometric coefficients of PP_i_ in the MGE pseudoreaction in millimoles per gram of MGE using the total molar weight of the MGE. The total molar mass of the MGE was calculated using the total mass of all genome and proteome components.

Finally, we formalized the single-direction pseudoreaction based on the previous calculations of stoichiometric coefficients, such that the sum across the stoichiometry for nucleotide content, amino acid content, ATP requirements, and H_2_O composed the left-hand side and the right-hand side summed across the stoichiometry of ADP, H^+^, and P_i_ generation via amino acid polymerization, plus PP_i_ generated by nucleotide polymerization. Reactions were added to our host *E. coli* metabolic model iJO1366 using COBRAPy. Initial upper and lower bounds on MGE production were set at 0 and 1,000, respectively, to match the typical FBA standard used to reflect the potential for unlimited flux.

### Simulation parameters

We used the COBRAPy package v. 0.29.0 (55) to perform all FBA, pFBA, and FVA simulations. Generally, we assumed that carbon was the only limiting resource and therefore ran simulations under the condition that all other nutrients were present in excess (Table S13). Lactose was used as the default carbon source for all experiments to replicate the conditions of our *in vitro* mutualistic system. To create a host-optimized state in our *E. coli* model, the objective function was set to the iJO1366 biomass function (BIOMASS_Ec_iJO1366_core_53p95M), with a lower bound of 0 on both plasmid-F128 and phage-M13 pseudoreactions. In a plasmid-F128-optimized state, the objective function was set to the plasmid-F128 biomass pseudoreaction with a lower bound of 0 mmol gDW^−1^ hr^−1^ on the phage-M13 pseudoreaction. In a phage-M13-optimized state, the objective function was set to the phage-M13 pseudoreaction with a constant lower bound of 0.9 mmol gDW^−1^ hr^−1^ on the plasmid-F128 pseudoreaction. Optimization was completed with the Gurobi optimizer.

dFBA was completed using COMETS v. 0.5.2 (56) to simulate the metabolic state of our strains in mono- or co-culture. Simulations were performed in a liquid environment with an initial population size of 1e-8 gDW for each species, which, based on an estimated cell dry weight for *E. coli* of 3e-13 grams (https://ecmdb.ca/e_coli_stats), corresponded to roughly 3.33 × 10^4^ cells per strain. The objective function of each species was set to its core biomass reaction. All non-carbon nutrients not provided via cross-feeding were expected to be present in excess, and the media were supplemented with 1,000 mmol per base nutrient; 2.78e-4 mmol carbon (as lactose) was included in all simulations, regardless of monoculture or co-culture condition. All media conditions used are available in Table S13. Simulations were performed for 200 cycles with a timestep of 1 hour. Species biomasses, media metabolite concentrations, and reaction fluxes were evaluated across the growth period (Fig. S1). The lower bound on plasmid production was set at 0.9 mmol gDW^−1^ hr^−1^ for both the F128+ state and F128+ M13+ state, while the lower bound on phage production in an F128+ M13+ state was set to 0.06 mmol gDW^−1^ hr^−1^. Host-optimized and intermediate carriage states for *E. coli ∆metB* were implemented as in COBRAPy. Optimization was completed with the Gurobi optimizer.

### FBA data analysis

Following completion of FVA, we identified reactions where the range of predicted flux values from either the MGE-optimized state did not overlap with the range of predicted flux values from a host-optimized state. Those reactions were then quantified as a percentage of all metabolic reactions in the system or as a fraction of the total number of reactions in the cellular subprocess to which they belonged. In addition, using pFBA, we calculated the parsimonious reaction flux for each cellular subprocess in terms of a fold-change as previously described (54). Briefly, the value can be calculated as:


$$\log_{2}\left(\frac{\sum_{i}F_{i}^{P}/\sum_{k}F_{k}^{P}}{\sum_{i}F_{i}^{H}/\sum_{k}F_{k}^{H}}\right)$$

where $\sum_{i}$$F_{i}^{P}$ is the sum of the parsimonious flux value through all reactions that belong to cellular subprocess *i* in an MGE-optimized state, $\sum_{k}$$F_{k}^{P}$ is the sum of the flux through all *k* reactions of the model in a MGE-optimized state, and the indexation *H* indicates those same values calculated when the model is in a host-optimized state. Calculations used absolute values to reflect the magnitude of changes in flux; the four reactions for which the direction of flux changed due to carriage (i.e., negative to positive or vice versa) were dropped from the metric. A positive log2(fold-change) value indicated higher demand for a subprocess in an MGE-optimized model, while a negative value indicated lower demand. Finally, we considered the dFBA-predicted metabolite concentrations in media from *E. coli ∆metB* with various carriage statuses growing in monoculture. Co-culture simulations were also performed in COMETS, and data are included in the supplement (Fig. S1).

### Bacterial co-culture system

Our bacterial obligate mutualism has been previously described (119, 120). The bacterial strains used for these experiments are listed in Table S14. Our *S. enterica* serovar Typhimurium LT2 has mutations in *metA* and *metJ* (121), causing it to oversecrete methionine. Our *E. coli* is auxotrophic for methionine due to a deletion of *metB* (119). Lastly, our strain of *M. extorquens* AM1 can get energy from C1 compounds but cannot assimilate carbon from these sources due to a deletion of *hprA* (120). To track individual species abundances during co-culture growth, we tagged *E. coli ∆metB* with a cyan fluorescent protein (CFP), *S. enterica* with a yellow fluorescent protein (YFP), and *M. extorquens* with a red fluorescent protein (RFP) (122). A strain of prototrophic *E. coli* MG1655 with the lac operon restored was also used in cross-feeding experiments with *S. enterica* in lactose minimal media, where MG1655 grows independently and *S. enterica* depends on carbon byproducts excreted from MG1655.

A fluorescent bacterial strain was used to confirm phage-M13 infection. *E. coli* CSH22 (*λ−*, *thi−, trpR−, ∆*[*lac-pro*]), an F′ strain belonging to the Cold Spring Harbor Laboratory collection, was transformed with the plasmid pJS001, which induces red fluorescence when phage-M13 particles enter the cell (43). This strain is referred to as JS002. Because CSH22 is a proline auxotroph, the F′ plasmid is required for growth in media lacking proline, while pJS001 carries an ampicillin resistance gene. Both plasmids can therefore be maintained in JS002 during growth in minimal media without proline and with added ampicillin. Additional strain and plasmid information is available in Table S14. Both strains were acquired from J. Shapiro.

### Mobile genetic elements of interest

The two primary MGEs used were the conjugative F128 plasmid and the filamentous phage M13. The plasmid F128 (*λ−, thr-1, araC14, leuB6*[Am], *lacY1*, *glnX44*[AS], *galK2*[Oc], *galT22*, ∆*trpE63, xylA5*, *mtl-1*, *thiE1*) is a ~230 kb fertility factor plasmid that was acquired from the Coli Genetic Stock Center at Yale University via J. Shapiro (123). The plasmid has a Tn*10* integration that allows the use of tetracycline to select for plasmid-positive cells. J. Shapiro provided a phage stock of phage-M13. Complete lists of gene products encoded on plasmid-F128 and phage-M13 are found in Table S7 and S15, respectively. Follow-up experiments were completed with a ~60 kb version of the conjugative F-plasmid consisting only of the conjugative machinery and the tetracycline resistance cassette called pOX38 (73). This plasmid was provided by P. Christie; gene product information is available in Table S6 (73). Gene products unique to each conjugative plasmid strain, either plasmid-F128 or pOX38, are found in Table S8.

Strains carrying plasmid-F128 were obtained via conjugation with an *E. coli* MG1655 plasmid-F128 donor that did not possess the lac operon. Colonies carrying both plasmid-F128 and phage-M13 were obtained by spotting six 10 µL spots of purified phage-M13 stock onto a top agar assay of exponentially growing cells that were already carrying the plasmid. Cells from one turbid clearing were scraped off the plate, resuspended in saline, and streaked onto an LB agar plate containing x-gal (5-bromo-4-chloro-3-indolyl-β-D-galactopyranoside) and 10 µg/mL of tetracycline. Phage infection of individual colonies was confirmed prior to use in experiments as described below. The same procedures were performed to obtain cells carrying pOX38 or pOX38 and phage-M13. The original pOX38 conjugative donor was an MC4100 *E. coli* strain provided by P. Christie (73).

### Media conditions

Minimal hypho liquid media was prepared as previously described (124). Each media component was sterilized prior to mixing at room temperature (124) (Table S16 and S17). As needed, solutions containing sulfur, nitrogen, phosphorus, calcium chloride, and metals were supplemented into each media type (Table S16 and S17), in addition to the appropriate carbon source and methionine/methylamine. In *E. coli ∆metB* monoculture conditions and in *E. coli ∆metB* and *S. enterica* cross-feeding media, the carbon source (lactose) served as the limiting nutrient. In *E. coli ∆metB and M. extorquens* and *E. coli ∆metB*, *S. enterica* and *M. extorquens* cross-feeding media, the amount of total nitrogen available in the medium (as methylamine, then metabolized by *M. extorquens*) limited total co-culture density. As a result, the final possible yield across different media types varied. These constraints were reflected in the media used for our dFBA simulations, as well. We performed routine culturing and plating of all bacterial strains with Miller Lysogeny Broth (LB) unless otherwise indicated; *M. extorquens* cannot grow on LB and was cultured on Difco Nutrient Broth (NB) when growth on rich media was required. When doing routine culturing of any strains carrying plasmid-F128 or pOX38 (including those with phage), 10 µg/mL of tetracycline was added to prevent plasmid loss. When culturing strains with pJS001, 100 µg/mL of ampicillin was added to prevent plasmid loss.

### Phage-screening assays

Phage infection confirmation assays were performed in 96-well flat-bottom plates on a Tecan Infinite Pro200 plate reader for 24 hours at 37°C with shaking at 432 rotations per minute. Individual phage-infected colonies streaked from plaques were resuspended from an agar plate into 200 µL of saline. 5 µL of each resuspended colony was then seeded into three wells containing 200 µL of LB and tetracycline media, in addition to two wells containing 200 µL of glucose and methionine hypho minimal media. 5 µL of a JS002 colony suspended in 200 µL of saline was also added to glucose and methionine wells along with phage-infected cells or into wells containing 200 µL of glucose and ampicillin hypho minimal media. Up to 10 *E. coli ∆metB* colonies were screened for infection at a time. After 24 hours of incubation, during which OD_600_ and red fluorescence measurements (Ex: 544, Em: 590) were taken every 20 minutes, JS002-only wells were used to establish an RFP baseline. Any wells containing both phage-infected cells and JS002 in which the RFP was at least greater than 1.5× times the RFP baseline were considered truly phage-M13-positive. The colony with the highest relative RFP was chosen for downstream experiments. LB wells seeded with cells from the chosen infected colony were then transferred into a flask containing 5 mL of LB and tetracycline and incubated for at least 30 minutes at 37°C with shaking at 120 rpm prior to addition in experiments.

### Community composition experiments

Community composition experiments were performed in 96-well flat-bottom plates on a Tecan Infinite Pro200 plate reader for at least 96 hours at 30°C with shaking at 432 rotations per minute. Experiments were terminated when all conditions reached the stationary phase. Overnight cultures in LB (*S. enterica, E. coli ∆metB*) or succinate and methylamine hypho minimal media (*M. extorquens*) were started from single colonies, diluted in fresh media, and grown to mid-log (~0.3 to 0.7 OD_600_) prior to being washed three times in saline and adjusted to a density of 10^7^ cells per mL. Relevant phage-M13-infected colonies were screened prior to addition to community composition experiments as previously described. Exponentially growing cultures of plasmid-carrying and phage-infected *E. coli* were grown to mid-log (~0.3 to 0.7 OD_600_), washed three times in saline, and adjusted to a density of 10^7^ cells per mL. Cells were used to inoculate 200 µL of appropriate medium with 3.0 × 10^5^ total cells per well (i.e., 3.0 × 10^5^ total *S. enterica* cells in monoculture; 1.0 × 10^5^ total *S. enterica* cells, 1.0 × 10^5^ total *E. coli*, and 1.0 × 10^5^ total *M. extorquens* cells in tripartite co-cultures). Starting cell densities were confirmed using CFUs at the start of the experiment on appropriate selective media.

We measured and recorded OD_600_, *E. coli ∆metB*-specific CFP (Ex: 430 nm; Em: 480 nm), *S. enterica*-specific YFP (Ex: 500 nm; Em: 530 nm), and *M. extorquens*-specific RFP fluorescence (Ex: 560 nm, Em: 610 nm) every 20 minutes. As previously described, we converted fluorescent protein signals to species-specific OD_600_ equivalents (125). Prior to community composition experiments, the linear relationships between optical density, species-specific fluorescence, and CFUs were validated for each *E. coli ∆metB* carriage status so that CFP and OD_600_ could be used to accurately assess biomass. Full factorial experiments testing all relevant monocultures and co-culture conditions were completed with three to five biological replicates per condition. To protect against edge effects, only water was placed in the outer ring of the 96-well plate. A minimum of two experiments per experiment type were set up and completed during different weeks unless otherwise indicated to confirm the repeatability of the results and protect against batch effects.

In experiments where one or multiple bacterial strains did not possess a fluorescent reporter, OD_600_ measurements were taken during growth, and relative bacterial densities were assessed using CFUs plated on appropriate selective media at the termination of experiments. Rates of plasmid loss were assessed at the end of experiments by plating *E. coli* from both mono- and co-culture conditions onto tetracycline plates in addition to antibiotic-free plates.

### Spent media preparation and metabolomic analysis

To generate spent media for metabolomic analysis, wild-type *E. coli ∆metB*, *E. coli ∆metB* carrying plasmid-F128, and *E. coli ∆metB* carrying both plasmid-F128 and phage-M13 were prepared in exponential cultures and washed three times in saline as previously described. Cells were seeded at an OD_600_ of 0.0005 in 50 mL of lactose and methionine hypho minimal media and allowed to grow at 30°C, shaking at 120 rpm until mid-log (~0.3 to 0.7 OD_600_). Cultures were spun down and then filter-sterilized using an Acrodisc 25 mm syringe filter with a 0.2 µm Supor membrane. Final CFUs of spent media cultures were assayed on appropriate selective media prior to spinning to determine cell density during mid-log growth. Three biological replicates were generated for each carriage status. 5 mL of spent media from each replicate for each condition was used to grow either *S. enterica* or *M. extorquens* (for *M. extorquens,* only growth in spent media from *E. coli ∆metB* carrying both plasmid-F128 and phage-M13 was completed). Partner strains were grown to the stationary phase, and cell culture media were filter-sterilized using an Acrodisc 25 mm syringe filter with a 0.2 µm Supor membrane. 1 mL of each condition (mid-log *E. coli ∆metB* spent media or stationary phase partner media) was pipetted into 1.5 mL microcentrifuge tubes and placed on dry ice. Samples were sent to Metware Biotechnology Inc. for widely targeted LC-MS.

UPLC-MS was performed by Metware Biotechnology Inc. using a Waters ACQUITY UPLC HSS T3 C18 column (1.8 µm, 2.1mm x 100 mm). The mobile phase used solvent A (0.1% formic acid in ultrapure water) and solvent B (0.1% formic acid in acetonitrile), with a gradient elution program of 95:5 vol/vol at 0 min, 10:90 vol/vol at 10.0 min, 10:90 vol/vol at 11.0 min, 95:5 vol/vol at 11.1 min, and 95:5 vol/vol at 14.0 min. The flow rate was 0.4 mL/min with a column temperature of 40°C and an injection volume of 2 µL. Metabolite detection was performed on a SCIEX QTRAP 6500 + mass spectrometer with an electrospray ionization (ESI) source in both positive and negative ion modes. Operating parameters included a source temperature of 500°C, an ion spray voltage (IS) of +5,500 V (positive) and −4,500 V (negative), and curtain gas (CUR) at 25 psi, ion source gas I (GSI) at 50 psi, and ion source gas II (GSII) at 50 psi. Collision gas (CAD) was high. 10 and 100 µmol/L polypropylene glycol solutions were used for instrument tuning and mass calibration in QQQ and LIT modes. Multiple reaction monitoring (MRM) was used to quantify metabolites with triple quadrupole mass spectrometry. Metware Biotechnology Inc.’s in-house metabolomics database was used for metabolite identification. A total of 589 unique metabolites were above the limit of detection in at least one condition; following signal drift and batch effect correction, 549 annotated metabolites with a coefficient of variation in quality control samples < 0.3 were retained for downstream analysis (Table S18) (126).

Metabolomics data, received from Metware Biotechnology Inc. as relative quantifications for each detected compound in each condition provided, were analyzed in two ways: principal components analysis and differential metabolite analysis. Prior to statistical analysis, metabolite intensities were pre-processed using biomass normalization (CFUs at the time of filter sterilization), a limit of detection threshold for missing values imputation (1/5 minimum observed value for a given variable), log2 transformation, and mean-centering (127–130). *E. coli ∆metB* mid-log samples were subsetted and used for principal components analysis. Identification of differential metabolites was completed in alignment with existing protocols (130–136), using univariate student’s t-tests and a supervised OPLS-DA model to assess differences between appropriate pairwise comparisons (134–136). Significance and overfitting of OPLS-DA models were evaluated using cross-validation and permutation testing as previously described (130, 134–136) (Table S19). Due to the limited number of samples in our data set, following confirmation of visible group separation with principal components analysis (137), we used a relaxed threshold of *pQ^2^* <0.1 to indicate model significance (Table S19) (130, 136). OPLS-DA was completed with the ropls v 1.26.4 in R. We also calculated the log2(fold-change) in intensity for each metabolite. Metabolites with a VIP >1 and a raw *P* value < 0.05 were considered significant. *P* values from univariate t-tests were also corrected using Benjamini-Hochberg correction for multiple comparisons; adjusted *P* values are reported alongside raw *P* values and VIP values in Table S3 and S5.

Spent media were prepared for HPLC assays as previously described in 5 mL volumes. For HPLC, 500 µL of each of three biological replicates generated from wild-type *E. coli ∆metB*, *E. coli ∆metB* carrying plasmid-F128, and *E. coli ∆metB* carrying both plasmid-F128 and phage-M13 were run on a Column Aminex HPX-87H at 46°C, flow 0.4 mL/min, solvent 1.5 mmol H_2_SO_4_, detector SPD-10A at 210 nm. Spent media data were analyzed using the R package chromatographR v. 0.7.0 for data pre-processing, alignment, and peak detection and integration. Concentrations of acetate and lactate in filtered spent media were estimated based on the linear relationship between peak area and mmol concentration using standards ranging from 0.39 mmol to 100 mmol. All spent media samples were stored at 4°C in light-protective boxes when not in use. Samples were analyzed by UPLC-MS or HPLC within 2 weeks of collection.

### Genome isolation and sequencing

Phage-M13 stocks were prepared for sequencing following ssDNA isolation. We incubated 200 µL of a high-titer phage lysate with 20 µL of DNase Buffer I 10× (Invitrogen), 2 µL DNase I (Invitrogen), and 0.4 µL RNase A (Qiagen) at 37°C for 1.5 hours without shaking. DNase I and RNase A were inactivated using 8 µL of 0.5M EDTA and a 10 minute incubation at 75°C. Next, we added 0.5 µL of Proteinase K (Invitrogen) and incubated the solution at 56°C for 1.5 hours without shaking. Finally, the phage ssDNA was purified using the DNeasy Blood and Tissue Kit (Qiagen), quantified on a Nanodrop, and sent to Plasmidsaurus (https://plasmidsaurus.com/) for standard whole plasmid sequencing. Our laboratory phage-M13 strain was found to be genetically identical to the “Rutgers” strain (GenBank: JX412914; Table S15) with two characterized variations: the non-synonymous T → G substitution at position 2095 that results in a phenylalanine to valine substitution in pIII, and the non-synonymous T → G substitution at position 6125 that results in an isoleucine to methionine substitution in pII (Table S10).

Plasmid-F128 and pOX38 were prepared for sequencing following dsDNA isolation from plasmid-carrying strains—the MG1655 donor for plasmid-F128 and *E. coli ∆metB* pOX38+ for pOX38. DNA was extracted from pelleted cells from an overnight culture using the DNeasy Blood and Tissue Kit (Qiagen), quantified on a Nanodrop, and sent to Plasmidsaurus (https://plasmidsaurus.com/) for standard bacterial genome sequencing. Plasmid-F128 was found to be nearly identical to the DHB4 plasmid F128-(DHB4) (GenBank: NZ_CP014271) and the tetracycline resistance genes from *Tn*10 (GenBank: AY528506), with the exception of the deletion of *ypjK*, nine non-synonymous substitutions in tetracycline resistance genes, and one non-synonymous substitution in *traD* (Table S7 and S10). Our strain of pOX38 was genetically identical to the MG1655 strain plasmid pOX38 (GenBank: NZ_OQ683454), though it does not possess the acyl-CoA reductase, acyl transferase, or long-chain-fatty-acid—CoA ligase genes found in NZ_OQ683454 and has a single synonymous A → T substitution at position 56,218 in *tetR* (Table S6 and S10).

## Data Availability

Data analysis, statistics, and figure generation were performed using R v. 4.2.1 using custom scripts available at https://github.com/bisesi/Phage-Community-Metabolism. Scripts for genome-scale metabolic modeling performed in Python 3.9.12 using COBRAPy and COMETSPy, along with raw experimental data for all detailed experiments, are available at the same link.

## SUPPLEMENTAL MATERIAL

Supplemental Figures msystems.00144-25-s0001.docx Figures S1 and S2.

Supplemental Tables msystems.00144-25-s0002.xlsx Tables S1 to S19.

ASM does not own the copyrights to Supplemental Material that may be linked to, or accessed through, an article. The authors have granted ASM a non-exclusive, world-wide license to publish the Supplemental Material files. Please contact the corresponding author directly for reuse.
