# Supplementary material for: Metabolic remodeling of microorganisms by mobile genetic elements alters mutualistic community composition
Source: mSystems. 2025 Aug 15;10(9):e00144-25. doi: 10.1128/msystems.00144-25 (PMC12455974; doi:10.1128/msystems.00144-25)
Supplement: Supplemental Figures — Figures S1 and S2. [file msystems.00144-25-s0001.docx]

SUPPLEMENTAL FILE 2

**
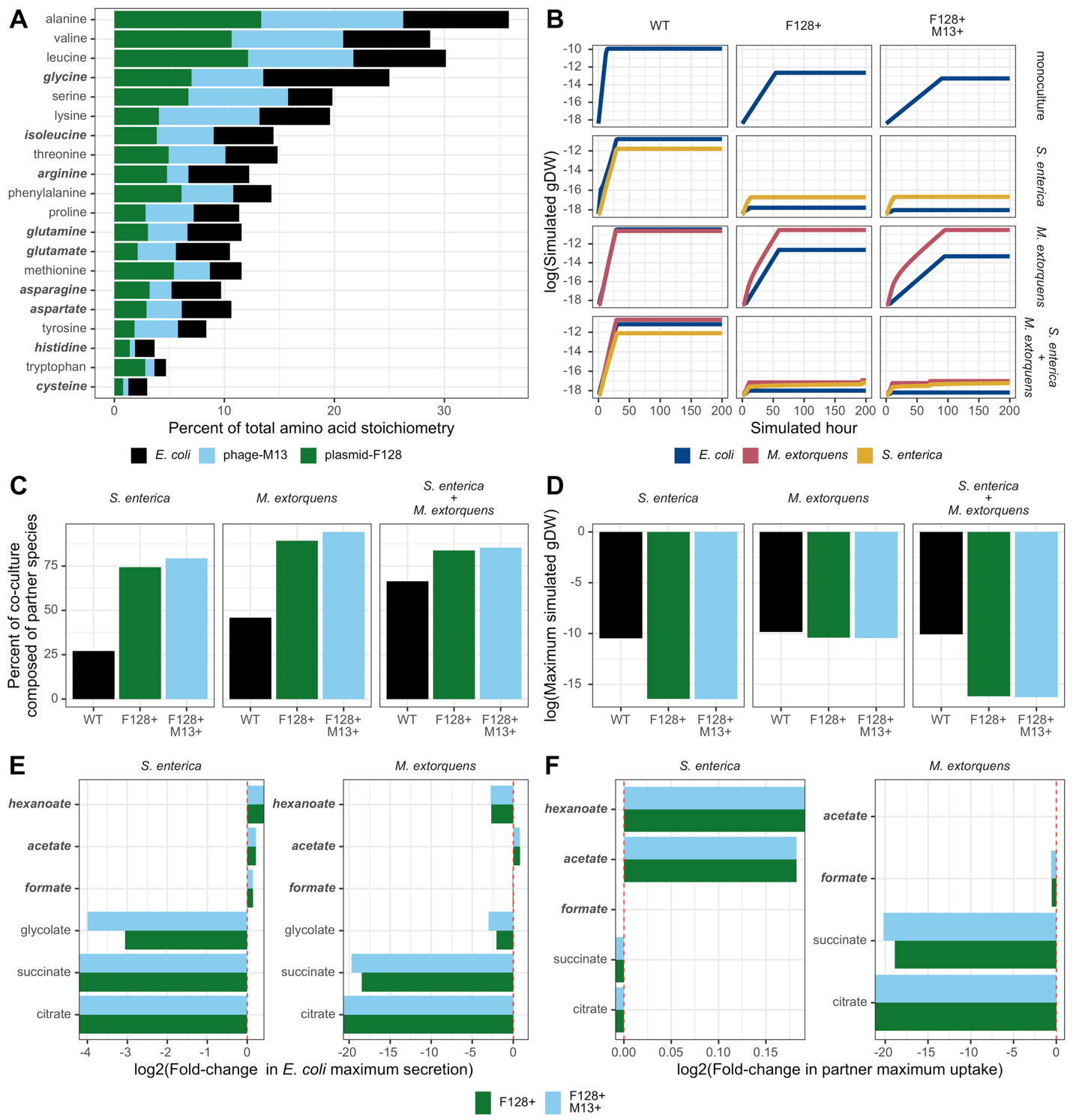
Figure S1. MGE carriage by *E. coli* changes dFBA predictions of composition and productivity in bacterial co-cultures. A:** Percent of total amino acid stoichiometry represented by each amino acid for host, plasmid and phage biomass reactions. Percents were calculated by dividing the stochiometric coefficient of a single amino acid by the sum of the stochiometric coefficients of all 20 amino acids for a given organism. Bolded and italicized amino acid names on the y-axis represent compounds that are a greater percentage of host stoichiometry than either MGE stoichiometry. **B:** Log-transformed dFBA-simulated growth, measured using gDW, for monocultures and two- and three-species bacterial communities grown with *E. coli ∆metB* with different carriage statuses. **C:** Percent of co-culture composed of partner species, calculated from species-specific gDW at the end of dFBA co-culture simulations. **D:** Log(Maximum total gDW) for co-cultures at the end of dFBA co-culture simulations, calculated from species-specific gDW. **E:** Log2(fold-change) in maximum secretion (exchange flux) of various metabolites by *E. coli ∆metB* during bipartite mutualistic growth with either *S. enterica* or *M. extorquens* in dFBA simulations. Compounds that were secreted more by *E. coli ∆metB* during carriage in at least one condition and were also taken up by either partner at a greater rate when growing with at least one type of MGE-positive *E. coli ∆metB* are bolded and italicized on the y-axis. **F:** Log2(fold-change) in maximum uptake (exchange flux) of various metabolites by *S. enterica* or *M. extorquens* during bipartite mutualistic growth with *E. coli ∆metB* in COMETS simulations. Compounds that were secreted more by *E. coli ∆metB* during carriage in at least one condition and were also taken up by either partner at a greater rate when growing with at least one type of MGE-positive *E. coli ∆metB* are bolded and italicized on the y-axis. *For all dFBA simulations, F128+ was modeled with a lower bound on plasmid production of 0.9 mmol gDW^-1^ hr^-1^, and F128+ M13+ was modeled with a lower bound on plasmid production of 0.9 mmol gDW^-1^ hr^-1^ and a lower bound on phage production of 0.06 mmol gDW^-1^ hr^-1^.*

**
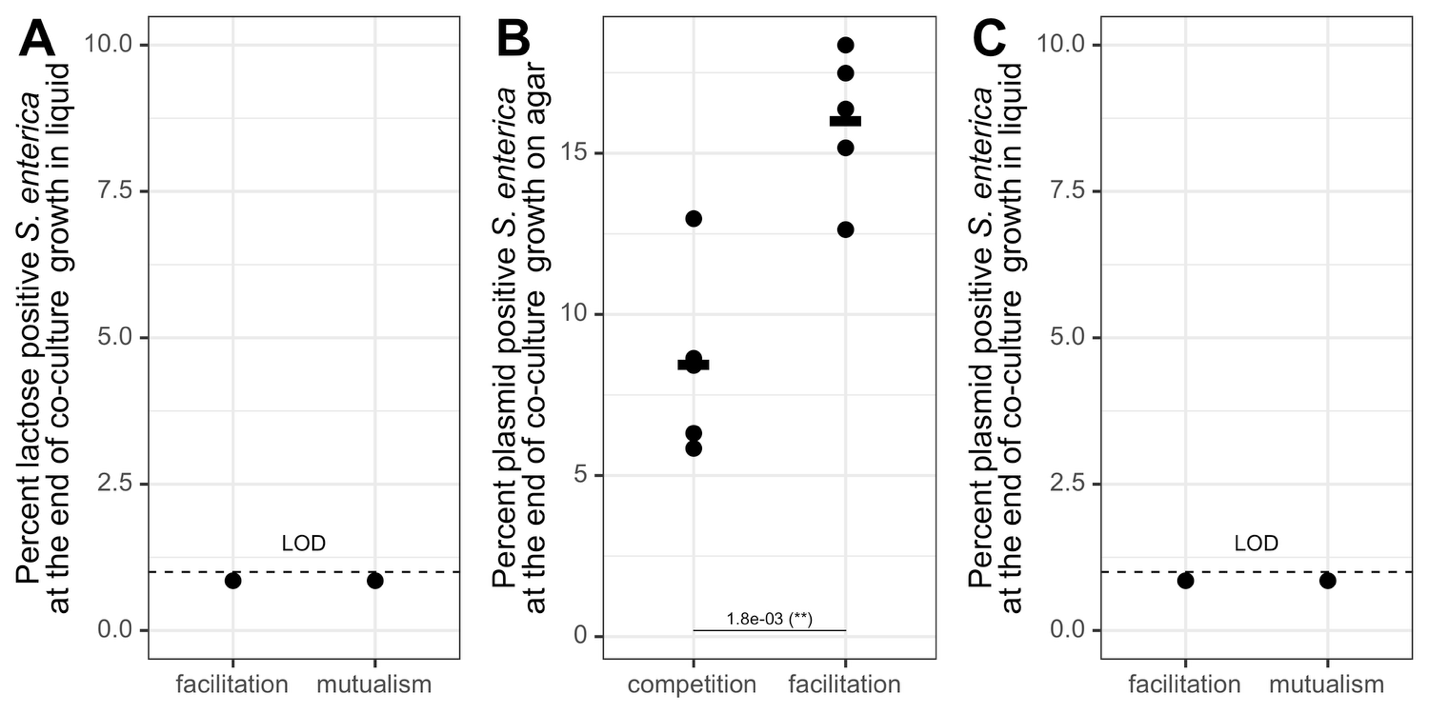
Figure S2. Conjugation of the F128 plasmid between *E. coli ∆metB* and *S. enterica* LT2 is possible on solid media but was not detected in mutualistic or facilitative experiments in liquid.** **A:** Lactose-positive *S. enterica* was not detected at the end of any co-culture experiments following growth with F128+ *E. coli ∆metB*. To test for *S. enterica* lactose utilization, half of the final co-culture volume following mutualistic growth between *S. enterica* and F128+ *E. coli ∆metB* (100µL) was serially diluted and plated on both lactose minimal hypho media plates with x-gal and lactose minimal hypho media plates with x-gal and 10µg/mL of tetracycline. Only *S. enterica* with growth enabled by carriage of plasmid-F128 would be able to grow on these plates. The limit of detection for lactose-positive cells was 1% of final *S. enterica* CFU/mL. **B:** Rates of conjugation between F128+ *E. coli ∆metB* and a wild-type *S. enterica* LT2 on agar plates varied depending on the nutrients provided. Facilitative growth on lactose and methionine minimal hypho media led to a higher rate of conjugation than competitive growth on glucose and methionine minimal hypho media. Conjugation experiments were performed by squirting 10µL of *E. coli ∆metB* F128+ directly on top of 10µL of a wild-type *S. enterica* LT2 on the surface of plates. Conjugation plates were incubated for 48 hours at 37°C before cells were scraped off plates into saline, pelleted, washed, serially diluted and plated at a volume of 100µL on either citrate minimal hypho media plates or citrate minimal hypho media plates with 10µg/mL of tetracycline. All *S. enterica* LT2 (but not *E. coli ∆metB* F128+) could grow on citrate minimal hypho media plates, while only *S. enterica* LT2 carrying plasmid-F128 could grow on citrate and tetracycline plates. The number of colonies counted on these plates were used to calculate rates of conjugation. Statistical significance was determined using a Welch’s t-test. Five replicates were completed per condition. The limit of detection for plasmid-positive cells was 1% of final *S. enterica* LT2 CFU/mL. **C:** There was no detected conjugation between *E. coli ∆metB* F128+ and a wild-type *S. enterica* LT2 in liquid media. Conjugation experiments were performed by adding 100µL of *E. coli ∆metB* F128+ to 100µL of a wild-type *S. enterica* LT2 in 5mL of liquid lactose minimal hypho media or lactose and methionine minimal hypho media. Conjugation liquid cultures were incubated for 48 hours at 37°C before cells were pelleted, washed, serially diluted, and plated at a volume of 100µL on either citrate minimal hypho media plates or citrate minimal hypho media plates with 10µg/mL of tetracycline. All *S. enterica* LT2 (but not *E. coli ∆metB* F128+) could grow on citrate minimal hypho media plates, while only *S. enterica* LT2 carrying plasmid-F128 could grow on citrate and tetracycline plates. The limit of detection for plasmid-positive cells was 1% of final *S. enterica* LT2 CFU/mL. Three replicates were completed per condition.
